# Singular Interface Dynamics of the SARS-CoV-2 Delta Variant Explained with Contact Perturbation Analysis

**DOI:** 10.1021/acs.jcim.2c00350

**Published:** 2022-06-06

**Authors:** Aria Gheeraert, Laurent Vuillon, Laurent Chaloin, Olivier Moncorgé, Thibaut Very, Serge Perez, Vincent Leroux, Isaure Chauvot de Beauchêne, Dominique Mias-Lucquin, Marie-Dominique Devignes, Ivan Rivalta, Bernard Maigret

**Affiliations:** † Laboratoire de Mathématiques (LAMA), Université Savoie Mont Blanc, CNRS, 73376 Le Bourget du Lac, France; ‡ Dipartimento di Chimica Industriale “Toso Montanari”, Universitá degli Studi di Bologna, Viale del Risorgimento 4, I-40136 Bologna, Italy; § Institut de Recherche en Infectiologie de Montpellier (IRIM), Université Montpellier, CNRS, 34293 Montpellier, France; ∥ Institut du Développement et des Ressources en Informatique Scientifique (IDRIS), CNRS, rue John von Neumann, BP 167, 91403 Orsay cedex, France; ⊥ CERMAV, University Grenoble Alpes, CNRS, 38000 Grenoble, France; # Inria, LORIA, University of Lorraine, CNRS, F-54000 Nancy, France; ∇ ENSL, CNRS, Laboratoire de Chimie UMR 5182, 46 allée d’Italie, 69364 Lyon, France

## Abstract

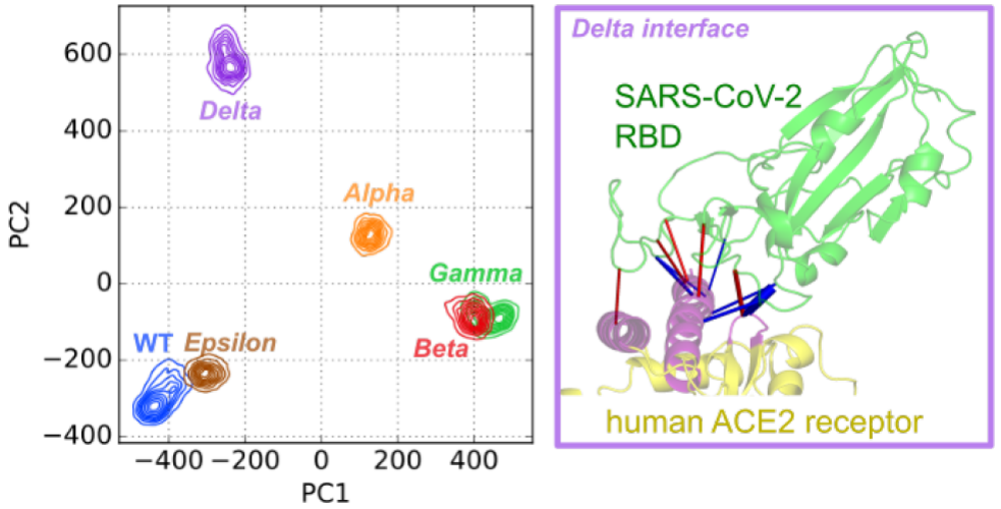

Emerging SARS-CoV-2 variants raise concerns about our ability to withstand the Covid-19 pandemic, and therefore, understanding mechanistic differences of those variants is crucial. In this study, we investigate disparities between the SARS-CoV-2 wild type and five variants that emerged in late 2020, focusing on the structure and dynamics of the spike protein interface with the human angiotensin-converting enzyme 2 (ACE2) receptor, by using crystallographic structures and extended analysis of microsecond molecular dynamics simulations. Dihedral angle principal component analysis (PCA) showed the strong similarities in the spike receptor binding domain (RBD) dynamics of the Alpha, Beta, Gamma, and Delta variants, in contrast with those of WT and Epsilon. Dynamical perturbation networks and contact PCA identified the peculiar interface dynamics of the Delta variant, which cannot be directly imputable to its specific L452R and T478K mutations since those residues are not in direct contact with the human ACE2 receptor. Our outcome shows that in the Delta variant the L452R and T478K mutations act synergistically on neighboring residues to provoke drastic changes in the spike/ACE2 interface; thus a singular mechanism of action eventually explains why it dominated over preceding variants.

## Introduction

The SARS-CoV-2 virus, associated with the Covid-19 pandemic, has spread all over the world by first infecting human pulmonary cells. This critical step is achieved through specific interactions between the homotrimeric transmembrane spike glycoprotein (S protein, with 1273 residues in each monomer) and human angiotensin-converting enzyme 2 (ACE2) receptors.^1,2^ This attachment to cells is specifically mediated by the receptor binding domain (RBD; residues 319–541) of the spike that binds with high affinity the N-terminal helix of ACE2,^3,4^ allowing subsequent conformational changes and fusion between cell and viral membranes. As in many other viral infectious diseases, the emergence of mutant strains (or variants) ineluctably has arisen due to its zoonotic origin, interspecies transmission, and human host adaptation. As the main important step in cell infection is the recognition of the specific ACE2 receptor, mutations occurring in spike protein may confer increased or decreased infectivity potential, contributing to changes in transmission rates. With the rapid emergence of variants of concern (VOC) that quickly spread worldwide, the characteristics of viral transmission, disease severity, and neutralization susceptibility have been compromised. The first variant of concern was identified in the U.K. in late December 2020 (Alpha variant, B.1.1.7 lineage). While another variant (Beta, B.1.351) emerged independently in South Africa, new variants arose in Brazil (Gamma, P.1), in California (Epsilon, B.1.427/B.1.429), and finally in India (Delta/Kappa, B.1.617.1/2/3). The Alpha and Epsilon variants were de-escalated as threats in summer 2021. In November 2021, the latest variant of concern (Omicron, B.1.1.529) was first detected in South Africa and soon spread to multiple countries, and it is now the current dominant form. Prior to this, the Delta variant was dominant for almost a year. The mechanisms by which these mutations modulate the infectivity or the severity of the disease are not fully understood, and only predictions can be drawn from phylogenetic studies^5^ or binding free energy calculations.^6^ With the focus on the first step of viral infection or cell entry, several mutations encountered in the spike RBD are commonly shared by most variants, such as N501Y and L452R. On the other hand, some mutations are more distinct, such as T478K, which was exclusive to Delta prior to the discovery of the Omicron variant. The physicochemical interactions between hydrophobic and charged residues might greatly alter the recognition phase or the binding affinity between RBD and ACE2 receptors. For instance, the mutation N501T has been already shown to reduce the affinity of host ACE2 protein and S protein *in vitro*.^7^

Here, we report an extensive investigation of the interaction of the spike RBD domain with its human ACE2 receptor at the atomistic level, for the original SARS-CoV-2 virus as well as its five variants that emerged in late 2020, as detailed in Table 1. To this aim, we focus on the analysis of the primary molecular interactions between spike and ACE2 based on experimental structural data available from the Protein Data Bank (PDB). First, we investigate contact changes between the available X-ray structures^3,18^ of wild type (WT) and the Alpha, Beta, and Gamma variants, at 2.85, 2.63, and 2.80 Å, respectively. Here, we did not compare those results with available cryo-EM structures of the Delta and Epsilon variants^19^ because the involved structures are resolved at a lower atomic resolution that does not allow appropriate computations of atomic contacts (i.e., >3 Å).

**Table 1:** SARS-CoV-2 Variants Investigated in the Present Work^a^

WHO label (lineage, PDB)	status	first detected	spike mutations	impact on transmissibility	impact on immunity	impact on severity	transmission in EU
Alpha (B.1.1.7, 7EKF)	DE^b^	U.K. (September 2020)	**N501Y**, D614G, P681H	yes^8^	no	yes^9,10^	low
Beta (B.1.351, 7EKG)	VOC	South Africa (September 2020)	**K417N**, **E484K**, **N501Y**, D614G, A701V	yes^11^	yes^12,13^	yes^9^	medium
Gamma (P.1, 7EKC)	VOC	Brazil (December 2020)	**K417T**, **E484K**, **N501Y**, D614G, H655Y	yes^14^	yes^15^	yes^9^	medium
Delta (B.1.617.2, none)	VOC	India (December 2020)	**L452R**, **T478K**, D614G, P681R	yes^16^	yes^16^	yes^16^	high
Epsilon (B.1.427/B.1.429, none)	DE^b^	USA (September 2020)	**L452R**, D614G	unclear^17^	yes^17^	no	very low

a The epidemiological status is as reported by the European Center for Disease Prevention and Control (ECDC) as of December 15, 2021 (https://www.ecdc.europa.eu/en/covid-19/variants-concern). Mutations of interest found in spike RBD compared to the WT SARS-CoV-2 strain are depicted in bold.

b DE, De-escalated.

In the fight against the Covid-19 pandemic, molecular dynamics (MD) simulations were particularly successful at guiding vaccine development,^20,21^ designing RNA polymerase inhibitors,^22^ investigating the binding of small molecules of the RBD,^23^ designing main protease inhibitors,^24^ and elucidating the role of glycans in SARS-CoV-2 viral entry.^25^ Notably, previous studies on the increased infectivity of variants investigated the role of mutations on antibody binding^26^ and uncovered an allosteric signaling between mutations in the Beta variant.^27^ In this work, we model all variants using a common modeling procedure starting from the WT structure with the highest resolution available at the time (PDB 6M0J), introducing *in silico* mutations and equilibrating structures. Then we perform MD simulations of the monomeric form (one unit of each protein) of various spike–ACE2 systems. Thus, we performed the analysis of the primary molecular interactions between spike and ACE2, focusing on the effect of the different mutations on the atomic contacts at the interface and the corresponding binding dynamics. This information is indeed not directly accessible from the crystallographic structural models available in the PDB (>200 X-ray or cryo-EM derived structures) and requires atomistic simulations. We adopted several tools to analyze the MD trajectories and to cross-compare them, including dihedral angle principal component analysis (dPCA),^28^ static and dynamical perturbation contact networks (PCN and DPCN, respectively), and contact principal component analysis (cPCA).^29^ The dPCA shows that the different mutations trigger similar rearrangements inside the spike RBD in the Alpha, Beta, Gamma, and Delta variants that are not fully reproduced in the Epsilon variant. Dynamical perturbation contact networks show that drastic differences in the interface dynamics arise between the Delta variant and the Alpha–Gamma group, despite the fact that these changes relate to mutations (L452R and T478K) that involve residues far from the interface. Finally, using cPCA, we show how synergistic effects of L452R and T478K mutations in Delta trigger a pattern of specific contact rearrangements that strongly affect the RBD/ACE2 interface. This knowledge on the initial molecular mechanisms triggered by the spike–ACE2 association provides a fundamental understanding of this critical aspect of viral infection and may be very valuable for the rational design of antiviral therapies.

## Materials and Methods

### Three-Dimensional Model Building and MD Simulations

#### RBD/ACE2 Wild Type and Mutant Complexes

Several similar structures of the RBD/ACE2 wild type human monomer–monomer complex are available in the PDB database^1,3,7^ (see Figure S1), and we used the one with the highest resolution (2.45 Å): 6M0J.^3^ The Visual Molecular Dynamics program (VMD)^30^ was used to prepare the structural models starting from the WT PDB structure and to introduce *in silico* mutations. Molecular dynamics (MD) simulations were performed with the NAMD package^31^ in conjunction with the recent CHARMM36 force field.^32^ Six RBD/ACE2 complexes were considered in the present work: the WT and five variants among the most infectious strains (Alpha B.1.1.7, Beta B.1.351, Gamma P.1, Delta B.1.617.2, and Epsilon B.1.427 variants). Each protein–protein complex was placed in a TIP3P^33^ water explicit solvent box of 150 Å^3^ with periodic boundary conditions to simulate the biological environment realistically. Next, Na^+^ ions were added to ensure neutrality of the periodic box. Each system was first energy minimized by performing 64 000 steps of conjugate gradient and next equilibrated (10 ns MD simulation), and a trajectory of 1 μs was then produced. The simulations were carried out in the isobaric–isothermal ensemble, maintaining constant pressure and temperature at 1 atm and 300 K, respectively, by means of Langevin dynamics and Langevin piston approaches as implemented in NAMD. The equation of motion was integrated every femtosecond, with the use of the r-RESPA algorithm^34^ to update short- and long-range contributions at different frequencies. Long-range electrostatic interactions were treated with the particle mesh Ewald approach.^35^ Every picosecond, one frame was saved from the trajectory file, leading to a total of 1 000 000 frames for further analysis.

### MD Analysis Tools

#### Root-Mean-Square Deviation

The root-mean-square deviation (RMSD) of atomic positions is a first rough indicator of simulation convergence. First, we align trajectories with respect to their initial conformation by minimizing the RMSD of backbone atomic positions. Then we report minimal RMSD fluctuations over time. Since, in our models, the spike RBD contains 229 residues and the ACE2 protein contains 603 residues, it is possible that averaging the RMSD on the global ACE2/RBD complex hides destabilization due specifically to mutations in the RBD. To assess more directly possible effects of mutations, we also compute the RMSD of backbone atomic positions restricted either to the RBD (excluding terminal segments, residues S325–N540) or to the receptor binding motif (RBM; residues S438–Q506) where most mutations are located.

#### Dihedral Angle Principal Component Analysis

Principal component analysis (PCA)^36−44^ of MD simulations is a general method to extract essential motions of a system and to reduce the high-dimensional evolution of a proteic system in a low-dimension landscape. Choosing appropriate features for PCA is crucial. External coordinates such as Cartesian coordinates have an intrinsic disadvantage over internal coordinates such as dihedral angles^28^ because they are not invariant by translation and rotation of the system and, thus, require an alignment of the system which may be imperfect, in particular, with a flexible system such as the ACE2 receptor. Moreover, a series of works show that using Cartesian coordinates PCA can only capture the general overall motions of a protein whereas dihedral angle PCA can capture both general overall motions and smaller internal motions, which leads to generally more accurate and well-resolved free energy landscapes.^45−47^ In this formulation, for each frame we compute 2*N* dihedral angles and linearize them from the circular space using the transformations
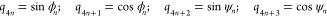
1with *n* = 1, ..., *N* corresponding to the *N* pairs of consecutive residues from which dihedral angles are considered (in practice = *N*_residues_ – *N*_chains_). In this study, we accounted for all ϕ and ψ backbone dihedral angles. Since RBD variants only show single point mutations, the considered models have all the same number of backbone dihedral angles and can be compared straightforwardly. An observation matrix *Q*_*i*,*j*_ of size *N*_frames_ × 2*N* is constructed, where the columns are all linearizations of ϕ and ψ dihedral angles and the rows are all possible observation states (10 000 frames for the WT and each variant, so 60 000 frames in total). The scikit-learn^48^ implementation of PCA decomposition to get the principal components (PCs) was used. With restriction to the two first eigenvectors, they can be used to obtain the free energy landscape of the system:
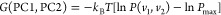
2

Here *P*(*v*_1_, *v*_2_) is the probability distribution obtained from a bivariate kernel density estimate,^49,50^ which is subtracted to ensure that Δ*G* = 0 for the lowest free energy minimum. Then the influence of the *n*th consecutive pair of residues in a component *i* is expressed as the sum of the squares of the influence of its features:
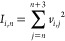
3where *v*_*i*_ is the eigenvector corresponding to component *i* and *v*_*i*,*j*_ is the coefficient corresponding to feature *q*_*i*,*j*_.

#### Ward’s Minimum Variance Method

Considering that the dPCA is built on the maximization of variance property, in order to find clusters of frames in the highest density regions of the projection, it is meaningful to group together minimum variance regions. Thus, Ward’s minimum variance method^51^ has been used to build a hierarchical clustering of the frames in the projected space. We then measure the discrete acceleration of the height of each consecutive cluster, and we set the optimal number of clusters as the one that maximizes this acceleration. The acceleration on the *x*-axis is shifted so that the initial acceleration value is for a number of clusters equal to two. The ensuing clustering of frames allows differentiation of regions with the highest density in the system energy landscape. Ward’s minimum variance method also provides a good way to detect key moments in a given simulation where the system undergoes large dynamical changes.

#### Perturbation Contact Network Analysis

Contact networks represent a protein as a collection of nodes, i.e., residues, that are connected by edges if those residues satisfy a contact condition. Here, in line with our previous works,^52−54^ the contact condition is achieved if at least one heavy atom from a residue is at a distance below 5 Å from another heavy atom in another residue. Edges between residues are then weighted by the total number of atomic contact pairs that satisfy this contact condition. Individual contact networks can be obtained from experimental PDB structures or from frames of MD simulations. “Static” contact networks are derived from a single experimental structure, while time-averaged networks of MD simulations correspond to dynamical contact networks. Then, in order to compare two contact networks (whether static or dynamical) and highlight contact differences between these structures, we subtract one from the other (formally, we subtract their weighted adjacency matrices). The differences between the two contact networks are visualized on the 3D model of the protein by assigning colors to the edges of the dynamical perturbation network according to the sign of the edges. Here, when we subtract the WT network from the mutant network, we assign the color red to a positive sign (i.e., stronger contacts in the mutant) and blue to a negative sign (i.e., stronger contacts in the WT). Finally, for visualization purposes, a weight threshold can be applied to select edges kept for display. Here, in line with previous works,^54^ using a heavy-atom network, we used an absolute threshold of 5 when explicitly mentioned. Isolated nodes after this process are also pruned to simplify the visualization. The main advantage of such a method is to get a direct and global view of all interactions resulting from chain motions and to allow the detection of subtle movements, including those occurring in loops.

#### Contact Principal Component Analysis

We report the weights of the contact networks of every frame in a matrix *C* of size *N*_frames_ × *N*_contacts_. If a contact is not present in one frame, its weight is simply put as zero. We use principal component analysis (cPCA for contacts) to extract the principal components. The PCs are each of size *N*_frames_ and represent the projection of the frames in this component. During the decomposition, we compute the (ordered) eigenvectors of the covariance matrix. Each of these eigenvectors corresponds to a principal component and is of size *N*_contacts_, thus representing a linear combination of all contacts in the system. We define a new type of contact network: the *i*th PC network (PC*i*N) in which nodes are the amino acids of the protein, edges are all contacts, and weights are the values of the contacts in the eigenvectors. Each of these eigenvectors also corresponds to an eigenvalue, which is representative of the importance of the principal component. By design, the eigenvalues in PCA and eigenvectors are ordered; thus the PCs decrease in importance with the component number. Similarly to dPCA, frames can be clustered by using Ward’s minimum variance method in the first principal components.

## Results

### Static Perturbation Contact Analysis

**Figure 1:**
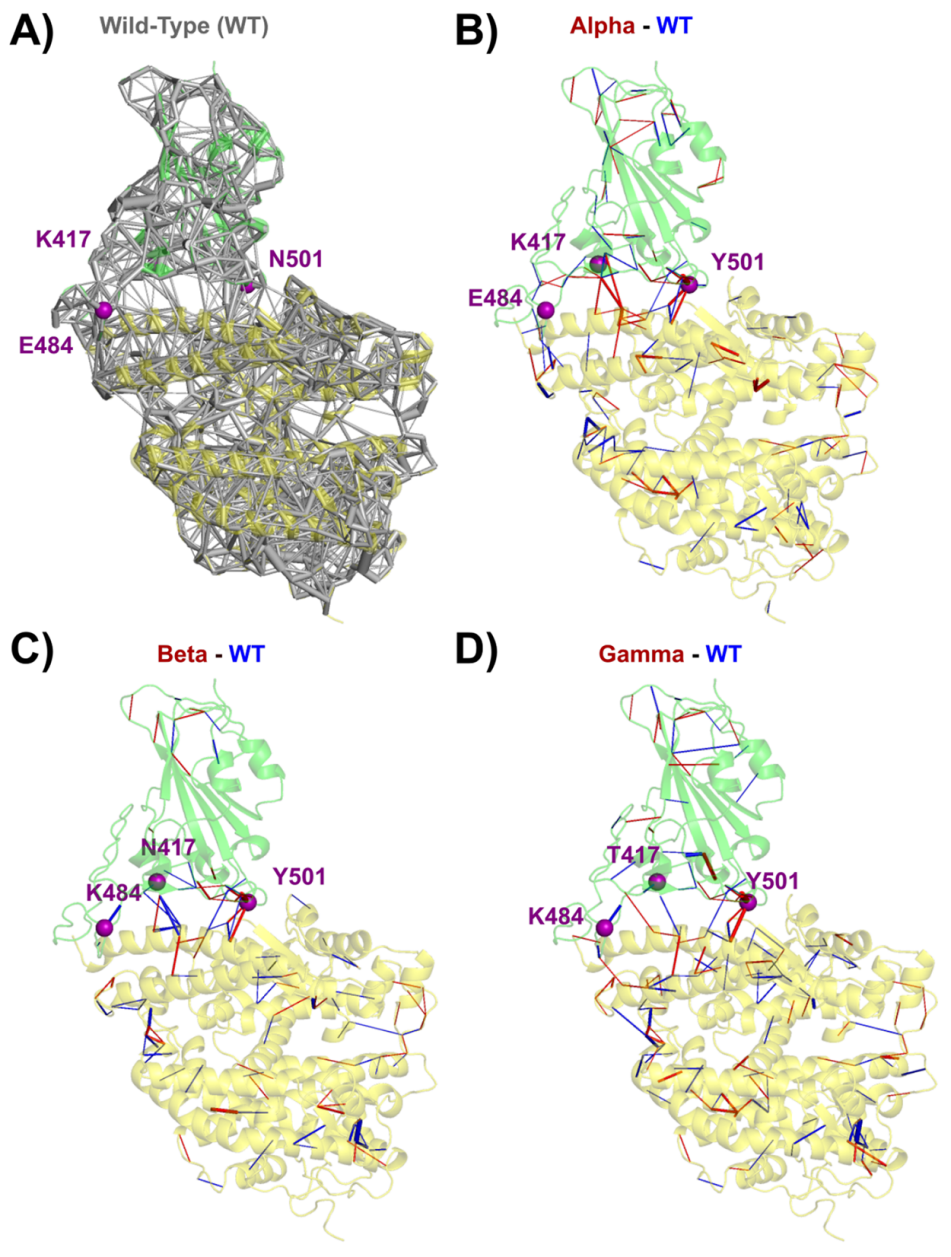
(A) Static amino acid network of the WT. (B–D) Static perturbation network, using the WT (PDB 6M0J) as reference network, at threshold 5 for (B) Alpha (PDB 7EKF, (C) Beta (PDB 7EKG), and (D) Gamma (PDB 7EKC).

Recently available structures of the Alpha, Beta, and Gamma variant RBDs in complex with the ACE2 protein^18^ give a precious molecular basis for the understanding of altered binding in emerging variants. In Figure 1B–D, we report the static perturbation contact network (PCN) between the RBD/ACE2 complexes from the Alpha, Beta, and Gamma variants (respectively PDB 7EKF, 7EKG, and 7EKC) and the WT (PDB 6M0J, Figure 1A), showing the main difference in atomic contacts deducible from X-ray experiments. Focusing on the WT, the interface between the spike RBD and the ACE2 involves various secondary structure elements in the spike RBD. First, in the α3 helix, residue K417 is in contact with residue D30 located in the α1 helix of the ACE2 receptor. Then, the α4−β5 loop (residues D442–Y451) has a few contacts with the α1 helix of ACE2 (i.e., G446–Q42 and Y449–D38). In the β5 sheet (residues L452–R454), residue Y453 is in contact with H34 of the ACE2 α1 helix. The β5−β6 loop (residues L455–F490) is also mainly in contact with the α1 helix (L455–H34, F456–T27, N487–Q24, Y489–F28, Y489–T27, Y489–K31), but some residues are also interacting with the α2 helix of the ACE2 receptor (N487–Y83, F486–L79, F486–Y83). In the β6 sheet (residues P491–Q493), residue Q493 is in contact with H34 and E35 of the ACE2 α1 helix. The nearby β6−α5 loop of RBD (residues S494–Y505) is also interacting with ACE2 α1 and with the β-turn (G352–D355), with the most relevant contacts being Q498–Y41, Q498–Q42, N501–Y41, N501–K353, and Y505–K353. The largest number of atomic contacts (i.e., 43 atomic pairs) in the WT is found for the interaction Y505–K353, while the N501–K353 and Q498–Y41 contacts are tied second (with 25 pairs). Among all mutated residues involved in the variants studied here, only K417 and N501 have a significant contacts across the interface (<5 atomic contacts) in the WT. It has to be noted that RBD residue E484 also possesses a minimal contact (one atomic pair) with K31 in the ACE2 α1 helix.

In the Alpha variant, which contains only the N501Y mutation, the main contact changes are directly associated with this residue, featuring an increase in contact between the Y501–Y41 and Y501–K353 pairs (+14 and +11 atomic pairs, respectively). These increases in contacts are partially compensated by some contact losses, including those of the Q498–Q42 (−7) and Y505–E37 (−5) interactions. Interestingly, far from the mutation spot, there is also an increase in contact with H34 in the ACE2 α1 helix associated with the Q493–H34 and the Y453–H34 interactions (+13 and +10 atomic pairs respectively, see Table 2) and some decrease in contact with the ACE2 α2 helix, involving F486–L79 (−5) and F486–Y83 (−4). Overall, the increase in number of atomic contacts of the Alpha variant with respect to WT is about 2%.

**Table 2:** Contact Values at Critical Residues in the Spike/ACE2 Interface in X-ray PDB Structures of the WT (PDB 6M0J) and Alpha (PDB 7EKF), Beta (PDB 7EKG), and Gamma Variants (PDB 7EKC)

	spike–ACE2														
	K417–D30	Y453–H34	L455–H34	F456–K31	E484–K31	F486–L79	F486–Y83	Q493–K31	Q493–H34	Q493–E35	Q498–Q42	N501–Y41	N501–K353	N501–G354	Y505–E37
WT	7	11	17	9	1	7	19	3	14	14	20	15	25	2	11
Alpha	6	21	16	10	1	2	15	11	27	0	13	26	39	2	6
Beta	0	14	11	12	0	9	18	10	7	2	14	28	35	2	4
Gamma	0	11	16	15	0	13	18	4	12	7	17	33	35	2	5

In the Beta variant, the same direct influence of the N501Y mutation is observed around residue N501. As expected, the K417N mutation breaks the K417–D30 salt bridge and contact losses are observed for K417–D30 (−7) and for nearby contacts: Q493–H34 (−7), Q493–E35 (−12), and L455–H34 (−6) pairs. A slight increase in contacts for the Q493–K31 pair (+8) partially compensates this effect. The other mutation, i.e., E484K, breaks the weak E484–K31 contact (−1). Overall, the Beta variant features a loss of about a 5% of contacts with respect to WT.

Finally, the Gamma variant is very similar to Beta but there the intensification of the Y501–Y41 contact is further magnified (15 atomic contacts in the WT, 26 in Alpha, 28 in Beta, and 33 in Gamma) while contact losses due to the loss of the K417–D30 salt bridge (T417–D30 also loses seven atomic contacts) are mitigated: only the Q493–E35 pair (−5) undergoes contact loss. Two other inter-residue interactions show some indirect effects of those mutations at the interface, i.e., F456–K31 (+6) and F486–L79 (+6). Similar to Alpha, the Gamma variant features a ca. 1% of contact increase with respect to WT.

More general trends of intradomain contact perturbations can be observed in the static PCN analysis, indicating that ACE2 contacts are more affected by mutations than RBD ones for the Alpha–Gamma variants, and overall, the Gamma variant features larger perturbations than the two others. The valuable information available from this static PCN analysis is however lacking dynamical effects that are going to be characterized in the following sections, where we also consider the comparison with the Delta and Epsilon variants that lack crystallographic structures with a resolution below 3 Å.

### Dihedral Angle Principal Component Analysis

We performed microsecond MD simulations on the WT and its five variants, Alpha–Epsilon, to characterize the effects of mutations on the RBD and ACE2 dynamics. RMSD analysis of these MD trajectories (Figures S2–S4) indicated that all systems equilibrated within 200 ns after the pre-equilibration steps, including the domains where most mutations are present, i.e., RBD and RBM. The dPCA has been initially performed on the whole (1 μs) MD simulation of each system (i.e., the concatenated values of backbone dihedral angles in all the frames for each system; see Figure S5). Because the ACE2 receptor is much more flexible than the RBD and to focus on dynamical changes in the RBD, we restrain the dPCA analysis to dihedral angles of the RBD. For each simulation, the PC1 and PC2 values undergo drastic adjustments between 200 and 600 ns. This indicates that some major rearrangements occur in the system, some of which can be attributed to the incorporation of *in silico* mutations. The latest of these important shifts occurs at 600 ns in the Gamma variant. Since this variant contains three different mutations (the most in any studied variant, tied with Beta), it is not surprising that it is the last to converge. Ward’s minimum variance method shows an optimal number of four, and each simulation remains in the same cluster during the last 400 ns. This indicates that our simulations have appropriately converged, and we can proceed with dPCA. Thus, here and in all the remaining analysis of this work, we focus on the frames of the last 400 ns for all MD simulations (employing then *N*_features_ = 722 and *N*_frames_ = 24 000).

**Figure 2:**
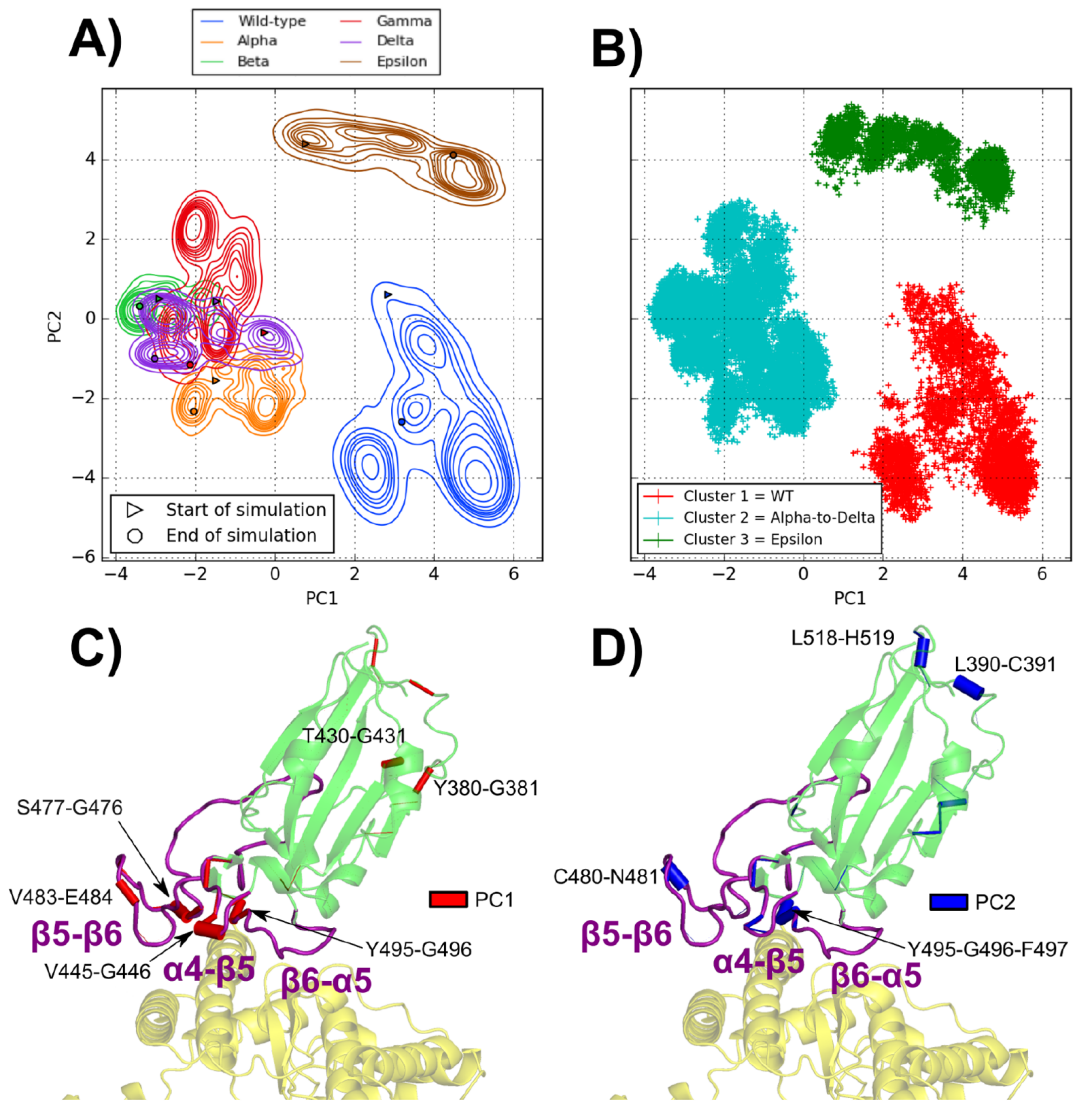
Projection of the frames corresponding to the final 400 ns of simulation for the six studied complexes in the two dPCA eigenvector dimensions with (A) contour plots representing a kernel density estimate of the population of each complex and (B) a scatter plot representing the three main clusters obtained through Ward’s minimum variance method. Representation of the influence (as cylinders with a width proportional to the influence) of each dihedral angle in the PC1 (C) and PC2 (D) eigenvectors on the spike RBD (green)/ACE2 (yellow) complex. The α4−β5, β5−β6, and β6−α5 loops are highlighted in purple.

When MD frames are represented in a PC1 vs PC2 plane, as depicted in Figure 2, the WT and Epsilon systems are both isolated (PC1 > 0 and PC2 < 0 for WT; PC1 > 0 and PC2 > 0 for Epsilon) from Alpha–Delta variants that are grouped together (PC1 < 0, PC2 ≈ 0). This grouping of Alpha–Delta variants as a function of the first two dPCA components suggests that different mutations might have similar effects on the RBD motion with respect to that of WT (see time evolutions of PC1 and PC2 in Figure S6D,E). In fact, the Delta variant does not share mutations with Alpha, Beta, and Gamma that, instead, all have in common the N501Y mutation. Notably, the Epsilon variant, despite sharing the L452R mutation with Delta, is separated from it (see also Figure S6D,E). The dPCA results indicate that PC1 (i.e., the largest variance axis) discriminates the Alpha–Delta group from both the WT and the Epsilon variant. Looking at the main conformational changes in the MD simulations, one can realize that the motion relating to WT and Epsilon (along PC1) refers to a large displacement of the α4−β5 loop (see Figure S15). On the other hand, the second principal component separates Epsilon from all the other systems, mainly because they feature different fluctuations of the β5−β6 loop (see Figure S16). Ward’s minimum variance method quantitatively confirms this behavior, showing an optimal number of clusters (see Figure S5) equal to three, corresponding to the WT, Epsilon, and Alpha–Delta groups. Interestingly, a previous study comparing the dynamics of SARS-CoV-2 and SARS-CoV (responsible for the SARS 2003 outbreak) evaluated that the increased rigidity in the β5−β6 loop of SARS-CoV-2 was linked to its higher infectivity because it enabled the formation of more stable bonds across the interface.^55^ This is in line with our results and suggests that the higher rigidity in the Alpha, Beta, Gamma, and Delta variant α4−β5 loops increases their transmissibility.

In Figure 2, the residue pairs with the most influence on the RBD dynamics are reported. The vast majority of these residues are located in three loops belonging to the RBM (438–506): α4−β5 (residues L455–F490), β5−β6 (residues L455–F490), and β6−α5 (residues S494–Y505). It is interesting to note that the α4−β5 and β6−α5 loops are in contact and contain respectively mutations L452R (Delta and Epsilon variants) and N501Y (Alpha, Beta, and Gamma variants). The time evolution of the V483–E484 dihedral angles (see Figure S8) actually shows that their fluctuations are analogous in variants with (Beta and Gamma) or without (Alpha, Delta, Epsilon) the E484K mutation. On the other hand, in the WT, these dihedrals have a different behavior, i.e., featuring larger fluctuations and significant shifts in the microsecond simulations. This suggests that, while the V483–E484 dihedral angle is involved in the main conformation motions of the RBD, the E484K mutation is not alone responsible for alterations of the RBM structure and motion. In fact, a previous study has uncovered an allosteric cross-talk between mutated residues K484 and Y501 mediated notably by N417^27^ in the Beta variant. Other sources of this cross-talk are found near the mutation spots in the β5−β6 and β6−α5 loops, precisely where our main dihedral changes in PC1 and PC2 are located. The present results suggest that, in the different variants, there are cross-talks between β5−β6 and β6−α5 which affect the loop flexibility. The above analysis of critical dihedral angles is therefore useful to understand the dynamics of the RBD upon mutations and to characterize some similarities and differences among various variants. However, dPCA does not provide an atomistic picture of the ACE2 and spike RBD protein responses to mutations. In order to recover this important information, an analysis of atomic contacts is reported in the next section, with a focus on the ACE2/RBD interface.

### Dynamical Perturbation Contact Network Analysis

**Figure 3:**
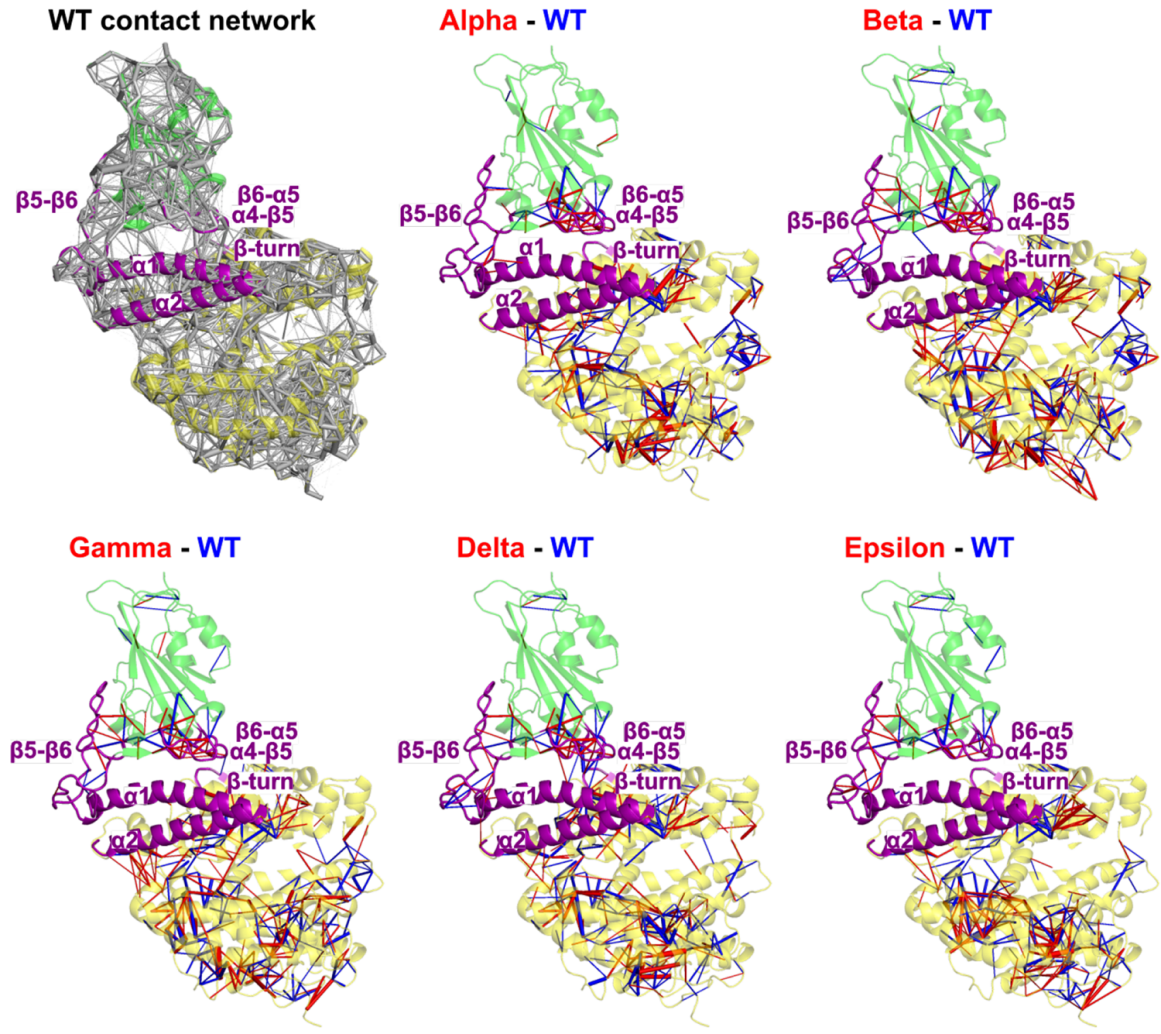
Complete perturbation network between each variant and the WT. The spike RBD (green)/ACE2 (yellow) complex is represented in cartoon representation. Stronger contacts are represented in the WT by a blue edge and in the variants by a red edge. Edge width is proportional to the weight.

The dynamical contact network of the WT simulation and dynamical perturbation contact network (DPCN) between variants and the WT are reported in Figure 3 (the individual amino acid networks are reported in Figure S9). At first glance, the resemblance between DPCNs from Alpha–Delta simulations is striking. Inside the spike RBD, there is one main patch of contact changes located between the α4−β5 and β6−α5 loops that is present in the Alpha–Delta variants, while a similar (but not identical) patch exists in the Epsilon variant. Interestingly, parts of the RBD located farther from the interface with ACE2 appear significantly less affected by mutations. The interface between the two proteins displays some contact changes, but with the notable exception of Delta, these are of a lesser magnitude (i.e., smaller number of total atomic contacts for each residue pair) than internal contacts perturbations in the RBD and in the ACE2 receptor.

Surprisingly, the ACE2 receptor is subject to much more contact changes than the RBD upon mutations, and some resemblance between contact perturbations can be observed among the five variants. This is consistent with studies showing that the ACE2 receptor is significantly flexible, in contrast to a high stability of the RBD/ACE2 interface.^56,57^ In particular, simulations of a ACE2 homodimer bound to the RBD show some conformations which may accommodate the binding of a single SARS-CoV-2 RBD to multiple ACE2 units. Looking at the propagation of perturbations within the ACE2 receptor, from the RBD interface to the opposite side of the ectodomain, one could speculate that, upon mutations in RBD, the binding of these five spike variants might eventually trigger a response of the ACE2 receptor that significantly differs from that of the WT, shifting the conformational ensemble of the RBD/ACE2 interaction toward RBD units binding to multiple ACE2 receptors.

Notably, when the total number of average contacts at the interface in the last 400 ns of MD simulations is considered (see Figure S11), all variants feature fewer atomic contacts at the interface than the WT. In particular, the interface between the ACE2 receptor and the Alpha and Beta variants shows a decrease of 12% in atomic contacts and the Gamma interface shows a decrease of 11%, while the Delta and Epsilon interfaces decrease by 4%. This is counterintuitive since we expect variants to show a higher RBD/ACE2 affinity, leading to an increase in contact count. In fact, experimentally, there is not a strict correlation between infective and transmissible variants and a higher affinity of the RBD/ACE2 complex.^58^ This suggests that variants use more complex mechanisms for cell entry and, in particular, a mechanism in which the RBD binds to more than one ACE2 unit is not predictable using our modeling. Therefore, the simplified mechanism described here at the RBD/ACE2 interface may be only the first step of a more complex mechanism in which the different variants facilitate the binding of the spike trimer to more than one ACE2 receptor (e.g., PDB 7V89 in the Delta variant). In fact, within this context, a slight destabilization of the monomeric RBD/ACE2 interface can be favorable to triggering RBD binding to multiple ACE2 receptors.

**Figure 4:**
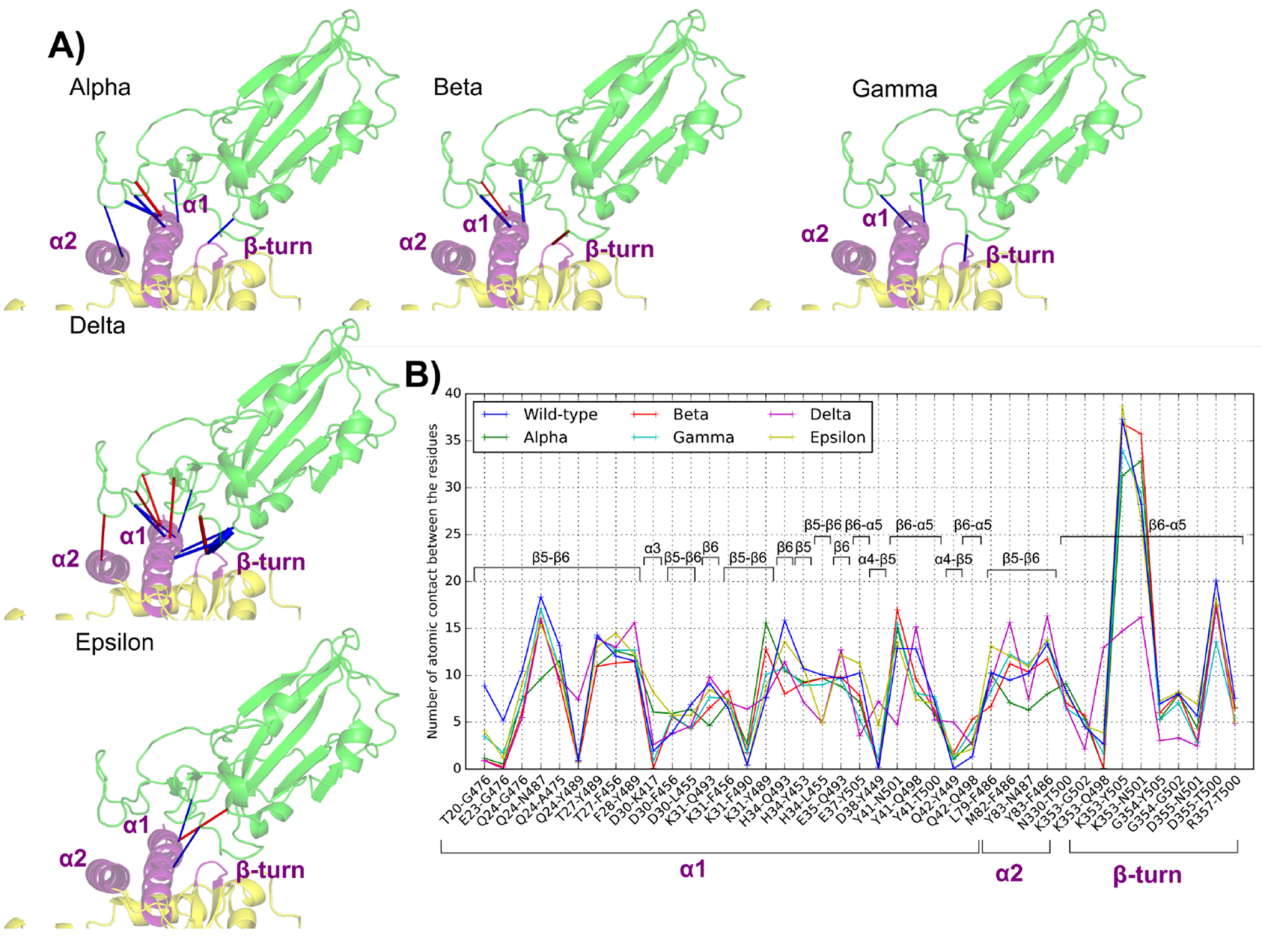
(A) Perturbation networks using a threshold value of 5 between the WT RDB (green)/ACE2 (yellow) complex and its mutants (Alpha, Beta, Gamma, Delta, and Epsilon). Stronger contacts are represented in the WT by a blue edge and in the variants by a red edge. Edge width is proportional to the weight and visualization factor, the same for each variant. (B) Average number of interresidual atomic contacts in all pairs at the interface (labeled in the WT residue name) with more than five contacts in at least one simulation.

In Figure 4, a close view of the DPCN near the ACE2/RBD interface is reported along with the list of contact pairs involved (Figure 4B). The spike RBD binds to three main areas of the ACE2 receptor: two helices, i.e., α1 (residues T20 to Y41) and α2 (mainly residues L79, M82, and Y83), and a β-turn (residues G352–D355). Among the WT residues mutated in the five variants, which are all located close to the RBD/ACE2 interface, only residues K417 and N501 are involved in the interface contacts during the MD simulation of WT, i.e., possessing (on average) >5 atomic contacts with ACE2. Although only two mutated residues are directly involved in the interface contacts, other atomic contacts at the interface are indirectly affected by mutations.

Here, we describe the direct and indirect contact perturbations upon mutations in the five variants. As shown in Figure 4A, the Delta variant is certainly the variant that features the largest number of interface contact perturbations despite the fact that, as described below, its mutations are not directly involved in interface contacts.

In the WT, the K353 residue in a β-turn of the ACE2 belongs to a dense interface contact network with the β6−α5 loop of the RBD (see the main peak in Figure 4B), involving the K353–N501 and K353–Y505 interactions. Notably, in the Delta variant, while N501 is conserved, these two contacts are disrupted and a new interface interaction is established between K353 and Q498. In the other variants, the K353–Y505 contact remains stable, but the K353–N501 interaction (stable in Epsilon) becomes slightly stronger in all N501Y variants, as a consequence of the π-cation formation mentioned above. Indeed, as discussed in the static PCN analysis, the K353–Y501 π-cation formation in the Alpha, Beta, and Gamma variants is accompanied by that of a T-shaped π-stacking interaction between Y501 and Y41, located at the α1 of ACE2. In contrast, in the Delta variant, the Y41–N501 contact is substituted by a stronger Y41–Q498 interaction.

In all models, the α2 helix of ACE2 is in contact with two residues of the RBD β5−β6 loop: F486 and N487. With respect to the WT, the Alpha variant features a slight decrease of all contacts in this region, while Beta and Gamma remain relatively untouched. The Delta variant shows again the most disparities: an increase in the M82–F486 and Y83–F486 contacts and a decrease in the Y83–N487 contact are detected. The proximity of these residues to mutation T478K suggests an indirect effect of this mutation (specific to the Delta variant) on the ACE2/RBD interface. In the Epsilon variant, just a slight increase in the L79–F486 contact is detected, and the rest of the contacts remain similar to those of the WT.

The α1 helix of ACE2 is in contact with many secondary structures of the spike. In particular, contacts with the β5−β6 loop of RBD involve the Y489 residue that features interesting contact perturbations upon mutations at the interface with ACE2 α1. In fact, Y489 strengthens the contact with residue F28 in WT while it establishes a new contact with residue Q24 in the Delta variant. In other variants, on the contrary, Y489–K31 is strengthened as a consequence of the loss of the weak E484–K31 electrostatic interaction found in the WT. Still, at the α1 helix (nearby K31), D30 establishes a salt bridge with residue K417, another mutation spot. This K417–D30 salt bridge has been found as a transient contact in MD simulations of the Epsilon and Alpha variants, but this interaction is never observed in the Beta and Gamma trajectories, featuring the K417N and K417T mutations, respectively. Surprisingly, in Delta and WT, without the K417 mutation, this salt bridge is also broken during the dynamics. While in the available X-ray structures (WT and Alpha) the K417–D30 salt bridge is present, our MD simulations suggest that this interaction might be actually weak and prone to rupture.

The Alpha–Gamma dynamics reproduce the main interface perturbation found in all corresponding crystal structures, which is the enhanced interactions between Y501, K353, and Y41. In Beta and Gamma, the contact loss associated with the K417(N/T)–D30 salt bridge breaking is also consistent with crystal structures. Interestingly, the WT dynamics shed light about the statistical significance of the K417–D30 interaction, since this salt bridge features a breaking-formation dynamic even in the absence of mutations.

Importantly, Delta mutation spots do not belong to the interface contacts, but they evidently have a significant impact at the interface. More generally, studying systematically the indirect effects of mutations is challenging, especially for comparative studies of mutants, and a more general type of analysis pointing at the most significant contact changes in various systems is required to understand why, for instance, the Delta variant features the largest interface perturbations despite the absence of interface mutations.

### Contact Principal Component Analysis

**Figure 5:**
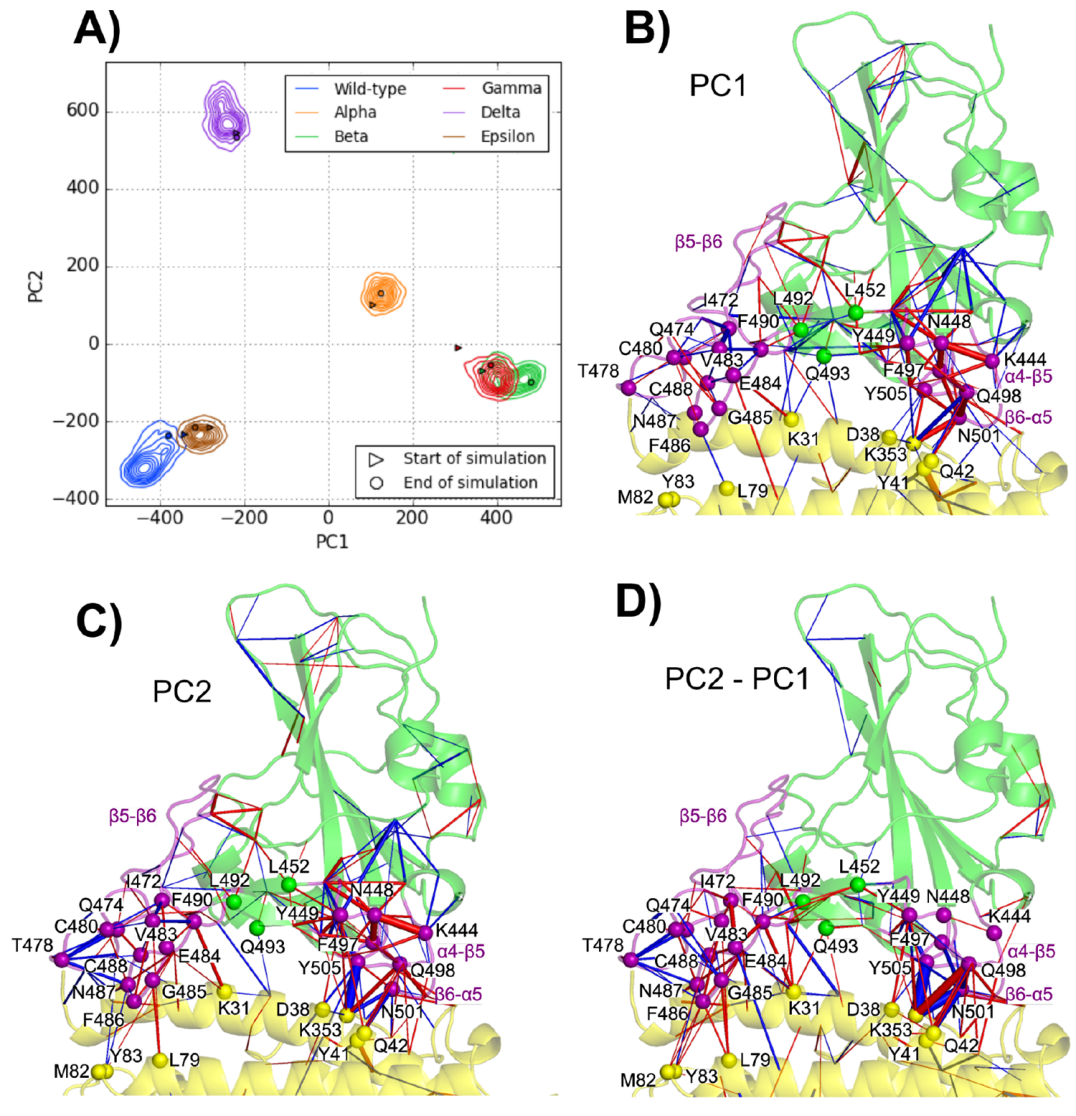
Projection of the frames corresponding to the final 400 ns of simulation for the six studied complexes in the two cPCA eigenvector dimensions, with (A) terrain lines representing a kernel density estimate of the population of each complex. Network representation of the influence (as cylinders with a width proportional to the influence) of each contact in the PC1 (B) and PC2 (C) and PC2–PC1 (D) eigenvectors projected on the spike RBD (green)/ACE2 (yellow) WT complex. Blue edges show a negative contribution to the principal component, while red edges show a positive contribution to the principal component. Contacts with a contribution of less than 1% to the eigenvector were discarded.

cPCA is used to characterize the overall information on dynamical contacts resulting from MD simulations of WT and RBD variants into their PCs. In particular, we found 9432 different contacts in the concatenated trajectories of the WT and five variants (considering the last 400 ns for each system). In Figure S7 we show that during the last 400 ns of each simulation PC1 and PC2 values are stable, which shows that our simulations have appropriately converged. As shown in Figure 5A, the scatter plot of the first two PCs shows how cPCA can cluster frames featuring similar dynamical contacts and thus characterize different systems according to that. In contrast to dPCA, here frames are separated into four main clusters: one with the WT and the Epsilon variant (negative PC1 and PC2), one with the Delta variant (positive PC2 and negative PC1), one with the Alpha variant (positive PC1 and PC2), and one with the Beta and Gamma variants (positive PC1 and negative PC2). In this representation, positive values of the PC1 separate Alpha, Beta, and Gamma from WT, Delta, and Epsilon. Positive values of the PC2, instead, discriminate Alpha and Delta from Beta, Gamma, Epsilon, and WT. The following PCs (see Figures S11–S14), i.e., those referring to smaller eigenvalues than the two largest ones, are associated with specific separations between systems: the third component separates the WT (negative PC3) and the Epsilon (positive PC3) from the rest, the fourth one separates Alpha (positive PC4) and Gamma (negative PC4) from the rest, and, finally, the fifth component discriminates between Alpha and Gamma (negative PC5) from Beta (positive PC5) and the rest. Smaller components than PC5 are associated with dynamical contact changes within simulations of each system; e.g., PC6 relates to dynamic contacts occurring in the Delta variant. In the dPCA, instead, this kind of clustering associated with each specific system starts with the third principal component. Thus, cPCA provides finer distinctions between the systems under investigation, in terms of dynamical contact changes, with respect to dPCA, especially showing some characteristics of the Delta variant.

The representations of PC1 (with positive values for Alpha, Beta, and Gamma and negative values for the rest) and PC2 (with positive values for Delta and Alpha and negative values for the rest) in terms of contact networks near the interface are depicted in Figure 5B,C. Therefore, in order to better differentiate the Delta network from the others, also the PC2–PC1 difference is represented in terms of contact network (see Figure 5D), with PC2–PC1 positive values being associated with the number of contacts that are large in the Delta variant and small in the Beta and Gamma variants, and vice versa for negative values of the PC2–PC1 difference (Alpha, Epsilon, and WT contribute only minimally to this network since PC2–PC1 differences are small in these cases). Here, the analysis of the PC2–PC1 differences provides insights into the link between the two Delta mutations (T478K and L452R) and their indirect effects at the interface.

**Figure 6:**
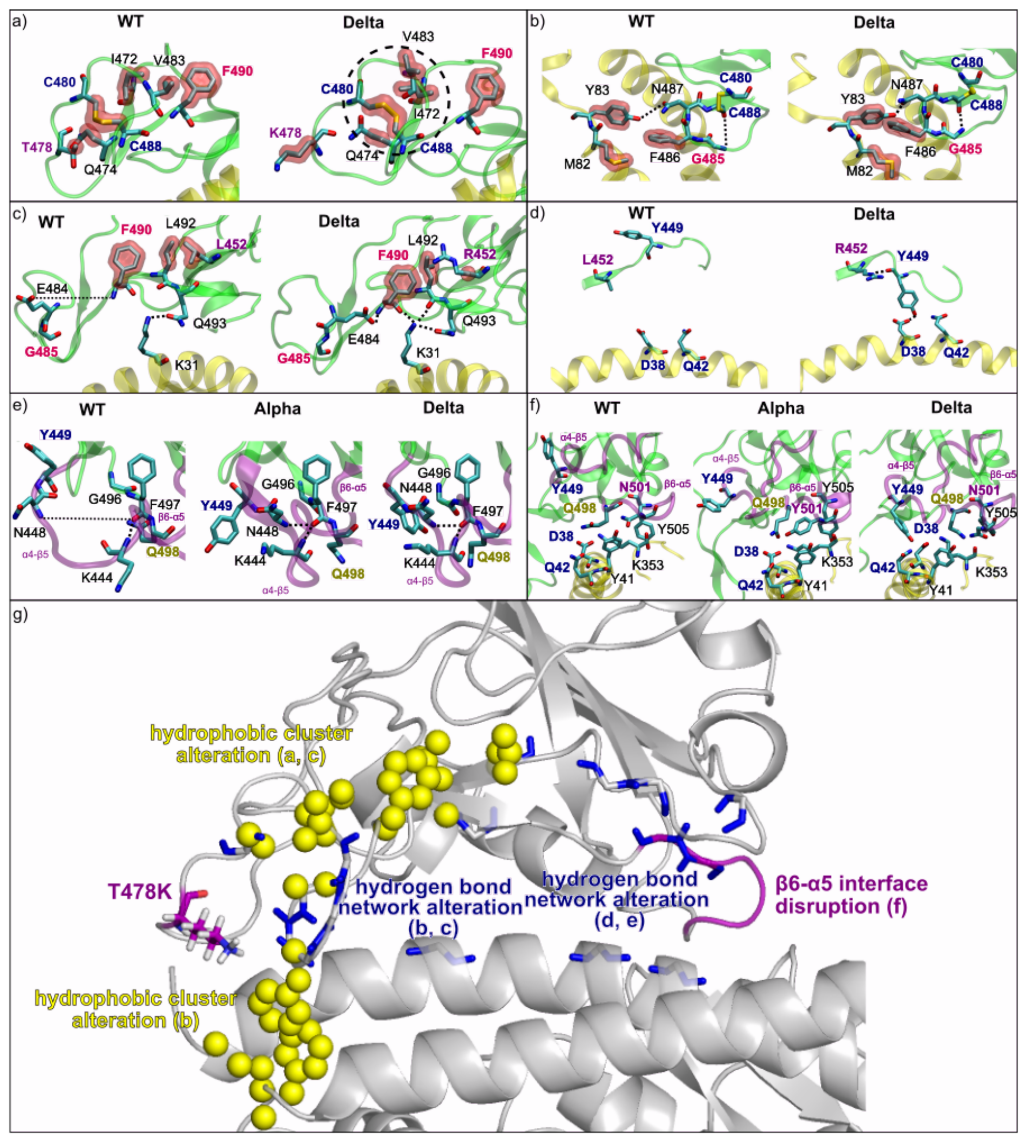
(a–f) Representative MD snapshots of some contacts in the different models emphasized by contact analysis. (g) Summary of the cross-talk between mutated residue T478 and the β6−α5 loop.

Starting from the T478K mutation (exclusive to Delta), located in the RBD β5−β6 loop, we found that this residue is a central hub of negative edges in both the PC2 and the PC2–PC1 networks (see Figure 5C,D), involving contacts with residues Q474, C480, F486, N487, and C488. This indicates that few contacts between those residues are characteristic of the Delta variant. In fact, as shown in Figure 6a, a hydrophobic cluster is observed nearby the C480–C488 disulfide bridge in the β5−β6 loop of the WT (and also in all other variants but Delta), involving the hydrophobic moieties of Q474 and T478 and residues I472, V483, and F490. Upon the T478K mutation, in the Delta variant, the insertion of the lysine side chain does not allow for such an arrangement and consequently the residue K478 is repelled out of the cluster. This loss of interaction in Delta is associated with flipping of the C480–C488 bridge that in turns pushes residue F490 far from the cluster. As a consequence of this rearrangement of the hydrophobic cluster, a backbone G485(NH)–C488(O) hydrogen bond is stabilized in Delta, determining a better folding of the β5−β6 loop, as depicted in Figure 6b. This differently folded structure also inevitably affects the dynamics of residues F486 and N487, which were previously highlighted in the DPCN of Delta at the RBD/ACE2 interface. These residues, indeed, show more contacts with M82 and Y83 (located in the ACE2 α2 helix) in the Delta variant than in the WT. This proves that the T478K mutation is indirectly responsible for the contact increase between the spike RBD and the α2 helix of ACE2.

The change in folding of the β5−β6 loop induced by the T478K mutation in Delta has, moreover, other indirect effects on the RBD/ACE2 interface that are synergetic with the effects of the L452R mutation. In fact, as shown in the PC2–PC1 network in Figure 6d, the negative edges around the T478K mutation in the RBD β5−β6 loop are somehow compensated by the positive edges around residue F490, i.e., the residue repelled out of the hydrophobic cluster in the Delta variant. This set of predominantly positive edges involves residues E484 (neighbor of G485), L492, L452 (mutated to R in Delta), and K31 across the interface. Indeed, the perturbations from T478K appear to be connected to those induced by the L452R mutation through residues F490 (in the β5−β6 loop) and L492 (in the β6 sheet). Figure 6c shows that the contemporary T478K and L452R mutations in Delta have a significant effect on the hydrogen bonding network around the interface residue K31. In particular, the dynamics of residue F490 is synergistically affected by the two mutations from two different sides: on one side the change in folding of the β5−β6 loop upon T478K mutation stabilizes the E484–F490 hydrogen bond while, on the other side, since F490 is also in hydrophobic contact with L492 and L452, upon L452R mutation, the arginine side chain promotes hydrogen bonding interactions of the L492 and F490 backbones with the K31 side chain. This finally results in three hydrogen bonds between the NH_3_^+^ head of K31 and the side chain oxygen of Q493 and the backbone oxygens of L492 and F490, which is a characteristic interface arrangement of the Delta variant (i.e., in the WT only Q493 is hydrogen bonded with K31), and it results from the combination of the two T478K and L452R mutations (far from the interface). Here, we should note that the Omicron variant also possesses the T478K mutations (same as Delta) but in conjunction with the E484A mutation. This latter mutation is somehow surprising since the E484K mutation is very common in spike’s mutants (e.g., it is found in Beta, Gamma, Mu, Lambda, Eta, and Theta) while E484A is exclusive to Omicron. This opens the question of how much the E484A mutation in Omicron could influence the effects of the Delta T478K mutations, which should be addressed in further studies. Notably, in the Beta variant, a cross-talk between mutated residue K484 and Y501 has been discovered.^27^ The present results suggest that, in the Delta variant, there is also an allosteric cross-talk between mutated residue K478 and the β6−α5 region (in which N501 is found). There is a possibility that the two cross-talks are incompatible with each other. It is worth mentioning that the E484K mutation, present in Beta and Gamma but not in Alpha, differentiates these variants in terms of the interface contacts between the ACE2 receptor and the RBD β5−β6 loop, involving the network of contacts highlighted in this region by the PC1 and PC2 components.

In the PC2–PC1 contact network (see Figure 6d), residue L452(R) is a bridging node that connects the contact perturbations in the β5−β6 loop (described above and involving the T478K mutation) with those of the α4−β5 and β5−β6 loops. Residue L452(R) has a positive edge with residue Y449 in this network, meaning that a close L452(R)–Y449 contact is typical of the Delta variant. In turn, Y449 displays direct connections with the interface residue, featuring positive edges with D38 and Q42 in the ACE2 receptor. Figure 6d shows that, indeed, upon the L452R mutation, the arginine side chain is able to make a hydrogen bond with Y449(O), which promotes a flipping of the Y449 side chain, allowing for the formation of a Y449–D38 interface hydrogen bond that alters the surrounding H-bonding network, involving also Q42.

Notably, the perturbations around residue Y449 in the PC2–PC1 network are minimal, as a consequence of the fact that perturbations inside the spike RBD (i.e., in the α4−β5 and β6−α5 loops) are rather similar in the PC1 and PC2 networks (see Figure 5B,C). In particular, the K444–N448 and N448–F497 pairs feature numerous contacts in PC1 and PC2, but they virtually vanish in the PC2–PC1 network, indicating that rearrangements of contacts in this region are significant in all variants but somehow differ from Epsilon that is more similar to WT, in line with the DPCN results depicted in Figure 3. At the same time, the largest PC2–PC1 differences are found at the interface between these spike RBD loops and the ACE2 receptor. Here, in the DPCN interface analysis we highlighted the role of residues Q498, N501, and Y505 in the β6−α5 loop in contact with Y41 in the α1 helix and K353 in the β-turn. Figure 6e shows how, in the Delta variant, upon the Y449 flipping mentioned above, the backbone N448(NH)–F497(O) hydrogen bond adds up to the preexisting K444(NH)–F497(O) one. Very interestingly, the very same two hydrogen bonds are also formed in the Alpha variant, featuring the sole N501Y mutation. This indicated that such a single mutation in the Alpha RBD creates a H-bonding network in the α4−β5 and β6−α5 loops of RBD similar to that produced by the indirect effects of the L452R mutation in the Delta variant (via residue Y449). Notably, these contact changes at the RBD common to both L452R and N501Y mutations in Delta and Alpha, respectively, have no effect on the interface contacts. In fact, as shown in Figure 6f, the WT and Alpha interfaces involve interactions between the same residues (i.e., D38, Y41, Q42, K353, Q498, N501(Y), Y505) despite the presence of the N501Y mutation, which only changes the type of some interactions (most notably the Y41–N501 π-polar interaction is promoted to a Y41–Y501 π–π interaction). On the other hand, the interface in the Delta variant largely differs from those of the WT and Alpha since the involved residues now include Y449 instead of N501 and Y505. Interestingly, this shows how the indirect effect of the Delta L452R mutation on the interface contacts, via the Y449 residue and the Y449–D38 interaction (see Figure 6d), has a large impact on the RBD/ACE2 interface as previously mentioned in the DPCN analysis; see Figure 4A. As a result of the L452R mutation in the Delta variant, thus, the formation of the R452–Y449 interaction is associated with structural rearrangements of the α4−β5 and β6−α5 loops that modify the interface contacts by including the Y449–D38 hydrogen bond and substituting the N501–Y41 interaction with the Q498–Y41 hydrogen bond, pushing residues N501 and Y505 away from the interface (breaking their contacts with residue K353). As is evident from Figure 5D, in fact, these interface changes are the most prominent in the PC2–PC1 network and represent the long-distance effects of the L452R mutation on the RBD/ACE2 interface.

## Conclusions

In this study, we first analyzed the (static) networks of atomic contacts between the spike RBD protein and the ACE2 human receptor based on the available crystallographic structures of the Alpha–Gamma variants of SARS-CoV-2, capturing the contact changes with respect to the WT and thus perturbations due to RBD mutations. Then, in order to account for dynamical effects of RBD mutations on spike/ACE2 interface contacts, microsecond MD simulations have been performed on the WT and the Alpha–Epsilon variants. Various tools for MD trajectories analysis have been used to recover the main similarities and differences between various spike RBD variants interacting with the human ACE2 receptor.

First, the analysis of protein essential motions based on backbone dihedral angles, namely dPCA, allowed recognizing mobile RBD regions whose dynamics is altered by mutations. The first principal components of backbone dihedral angles are associated with motions in the α4−β5 loop, while the second principal components are associated with motions in the β5−β6 loop. Considering these essential motions, three distinct behaviors have been observed for the various MD simulations: a cluster involving the Alpha, Beta, Gamma, and Delta variants features a tight α4−β5 loop and a flexible β5−β6 loop; the WT features a flexible α4−β5 loop and a tight β5−β6 loop; for the Epsilon variant, the tightest β5−β6 loop was observed along with a partially flexible α4−β5 loop. Interestingly, this clustering correlates with the impact in transmissibility and severity of the SARS-CoV-2 disease in the studied variants. These results suggest that the L452R and N501Y mutations have closely related effects on RBD motions near the interface. However, as evidenced by the dPCA of the Epsilon variant, these motions are not fully reproduced in the absence of the T478K mutation, which indicates an interdependence between these mutations. In fact, this change in flexibility of the RBD near the interface may be a first step facilitating the spike trimer binding to than one ACE2 receptor. Still, the dPCA analysis did not allow differentiating the Delta variant, the dominating one in most of 2021, from the others.

Then, we were able to recover some specificity of the Delta variant by studying the dynamical perturbation contact network, with a focus on the RBD/ACE2 interface. The comparisons between the WT atomic contact network and those of the Alpha–Epsilon variants showed many similarities among the Alpha–Gamma variants that share the N501(Y) mutation, which promotes specific perturbations for the interface contacts of Y501 with K353 and Y41 residues, while the rest of interface contacts remain essentially preserved. By contrast, in the Delta variant, significant contact changes at the interface have been found despite the absence of interface mutations. Indeed, all interface contact changes in Delta cannot be directly attributed to the T478K and L452R mutations that must have indirect (but large) effects on the interface.

The subsequent cPCA analysis shed, finally, light on the propagation of contact perturbations induced by the T478K and L452R mutations in the Delta variant. This analysis showed that the T478K mutation alters the contacts of a hydrophobic cluster (involving residues Q474, T478, I472, V483, and F490) around the C480–C488 disulfide bridge inside the β5−β6 loop of the RBD and promotes the formation of a G485–C488 backbone hydrogen bond. In turn, this rearrangement affects the positions of residues F486 and N487 that increase their interface contacts with the α2 helix of ACE2. At the same time, in the WT residues F490, L492, and L452 are involved in another hydrophobic cluster that upon L452R mutations is adjusted because of both the presence of residue R452 and the alteration of F490 contacts due to the T478K mutation. In turn, residues F490 and L492 create a triple hydrogen bond at the interface of Delta with residue K31, which was H-bonded just to residue Q493 in the WT. Since it belongs to both the aforementioned T478- and L452-related hydrophobic clusters, as a result residue F490 is central to the propagation of contact changes due to the simultaneous T478K and L452R mutations that result in cooperation in inducing the interface perturbations found in Delta.

Our results highlight the singular mechanism of action of the mutations in the Delta variant that could eventually explain why it dominated over preceding variants. Moreover, since the recent Omicron variant possesses the same T478K mutation but in conjunction with the E484A one, it remains to be elucidated if a synergistic long-range effect of multiple mutations such as that found here for the Delta variant is also operating for the currently dominating Omicron.

## Data and Software Availability

Software to compute DPCN, dPCA, and cPCA are available at the following github repository: https://github.com/agheeraert/pmdlearn. Molecular dynamics simulations of all RBD/ACE2 complexes are available upon request.

## Supporting Information Available

RMSD fluctuations, dPCA and cPCA on the complete trajectory, time evolution of key distances and dihedral angles, Cartesian coordinates PCA, cPCA components up to eight,, time evolution of interface loop flexibility (PDF)
