# Supplementary material for: Singular Interface Dynamics of the SARS-CoV-2 Delta Variant Explained with Contact Perturbation Analysis
Source: J Chem Inf Model. 2022 Jun 6;62(12):3107–22. doi: 10.1021/acs.jcim.2c00350 (PMC9199437; doi:10.1021/acs.jcim.2c00350)
Supplement: Supplementary file 1 — ci2c00350_si_001.pdf [file ci2c00350_si_001.pdf]

Supporting Information to Singular Interface Dynamics of the SARS-CoV-2 Delta Variant Explained with Contact Perturbation Analysis

Aria Gheeraert,[†,‡] Laurent Vuillon,[†] Laurent Chaloin,[¶] Olivier Moncorgé,[¶] Thibaut Very,[§] Serge Perez,[‖] Vincent Leroux,[⊥] Isaure Chauvot de Beauchêne,[⊥] Dominique Mias-Lucquin,[⊥] Marie-Dominique Devignes,[⊥] Ivan Rivalta,[*,‡,#] and Bernard Maigret[*,⊥]

†*LAMA, Univ. Savoie Mont Blanc, CNRS, LAMA, 73376 Le Bourget du Lac, France*

‡*Dipartimento di Chimica Industriale "Toso Montanari", Università degli Studi di Bologna, Viale del Risorgimento 4, I-40136 Bologna, Italy*

¶*Institut de Recherche en Infectiologie de Montpellier (IRIM), Univ. Montpellier, CNRS, 34293 Montpellier, France*

§*CNRS - IDRIS, rue John von Neumann BP 167 91403 Orsay cedex - France*

‖*University Grenoble Alpes, CNRS, CERMAV, 38000 Grenoble, France*

⊥*University of Lorraine, CNRS, Inria, LORIA, F-54000 Nancy, France*

#*ENSL, CNRS, Laboratoire de Chimie UMR 5182, 46 allée d'Italie, 69364 Lyon, France*

E-mail: i.rivalta@unibo.it; bernard.maigret@loria.fr

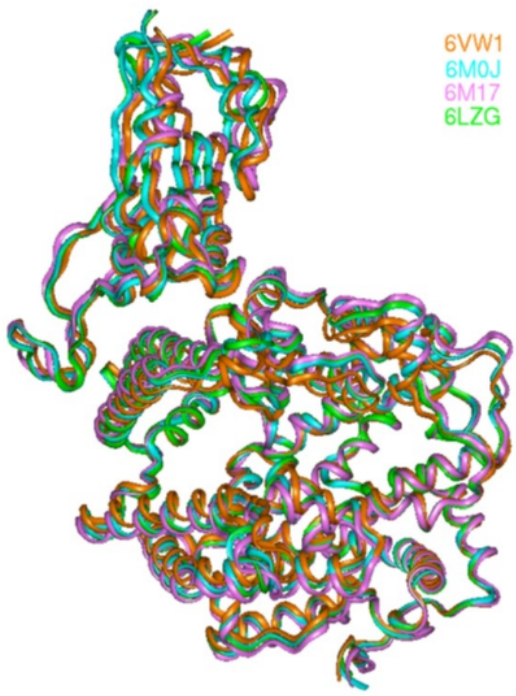

Figure S1. Superposition of four RBD/ACE2 complexes found in the PDB (PDB: GVW1, 6M0J 6M17 and 6LZG)

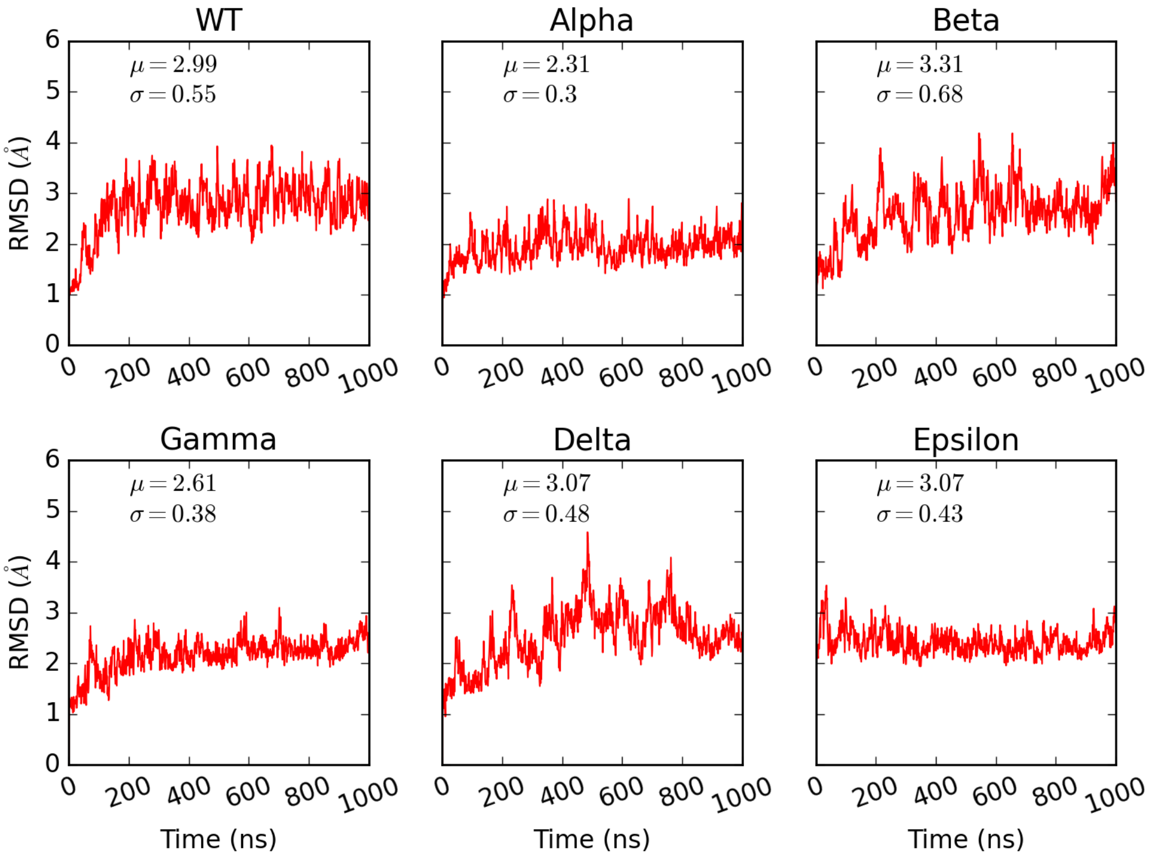

Figure S2. RMSD fluctuations during the microsecond MD simulations obtained for the wild type and five different variants of RBD/ACE2 complexes. For each complex, the RMSD were calculated only for the backbone atoms and excluding terminal loops (T27-D597 for ACE2 and S325-N540 for spike-RBD) and using the first frame of the simulation as reference.

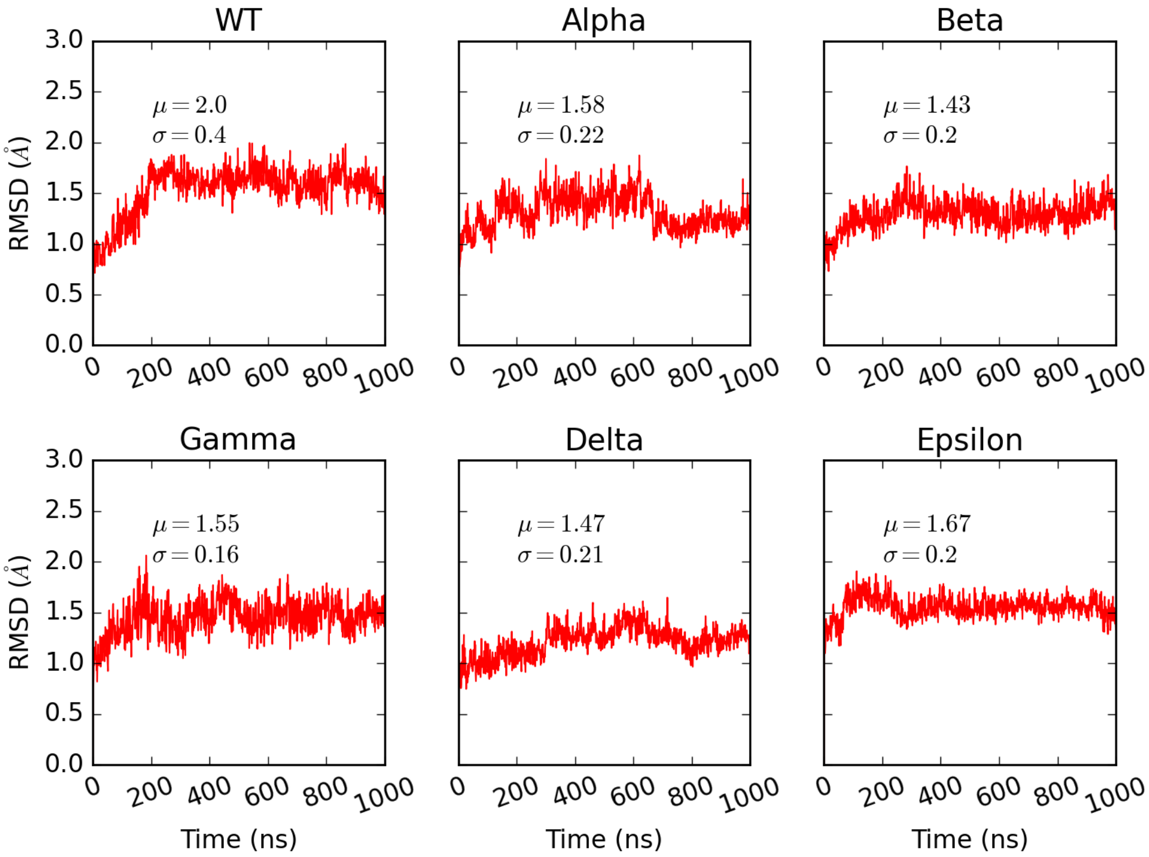

Figure S3. RMSD fluctuations during the microsecond simulations obtained for the six complexes. In each complex, the RMSD were calculated only for the backbone atoms of the RBD excluding terminal loops (residues S325-N540) and the initial frame as reference.

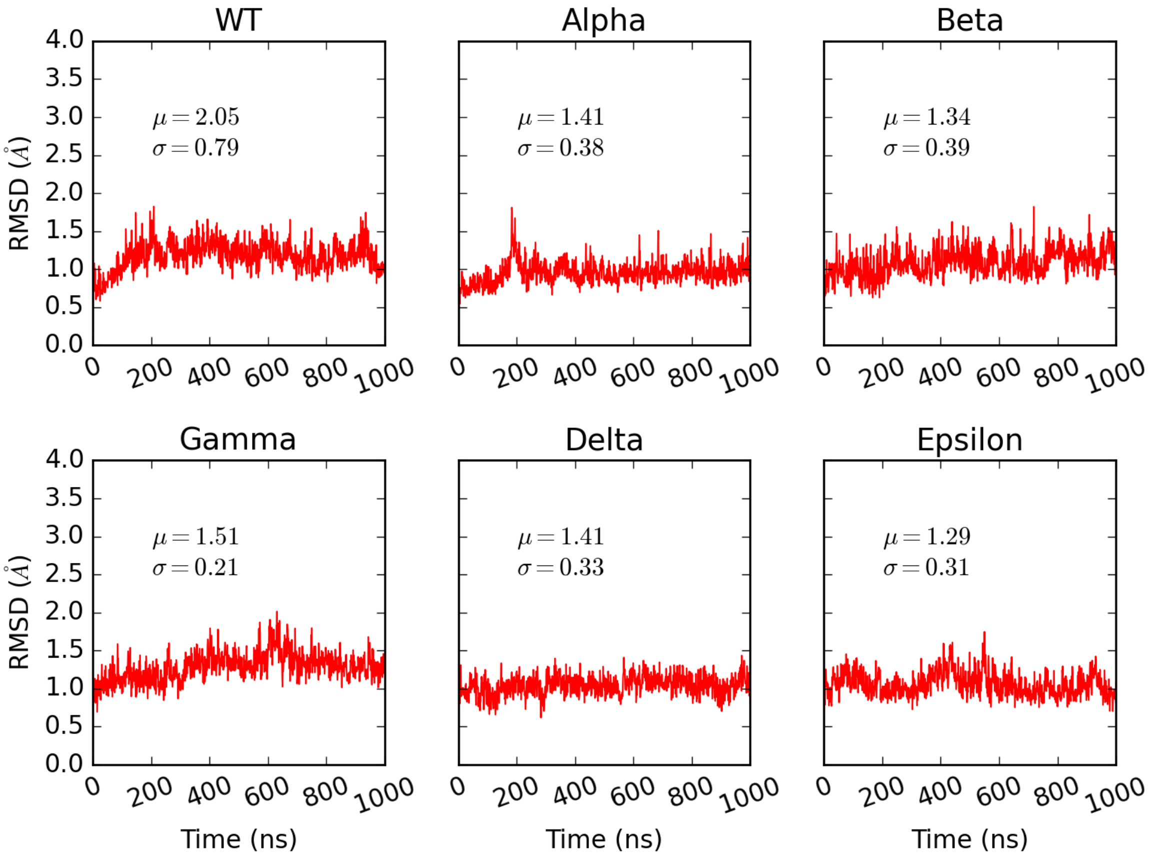

Figure S4. RMSD fluctuations during the microsecond simulations obtained for the six complexes. In each complex, the RMSD were calculated only for the backbone atoms of the RBM (residues S438-Q506) and using the initial frame as reference.

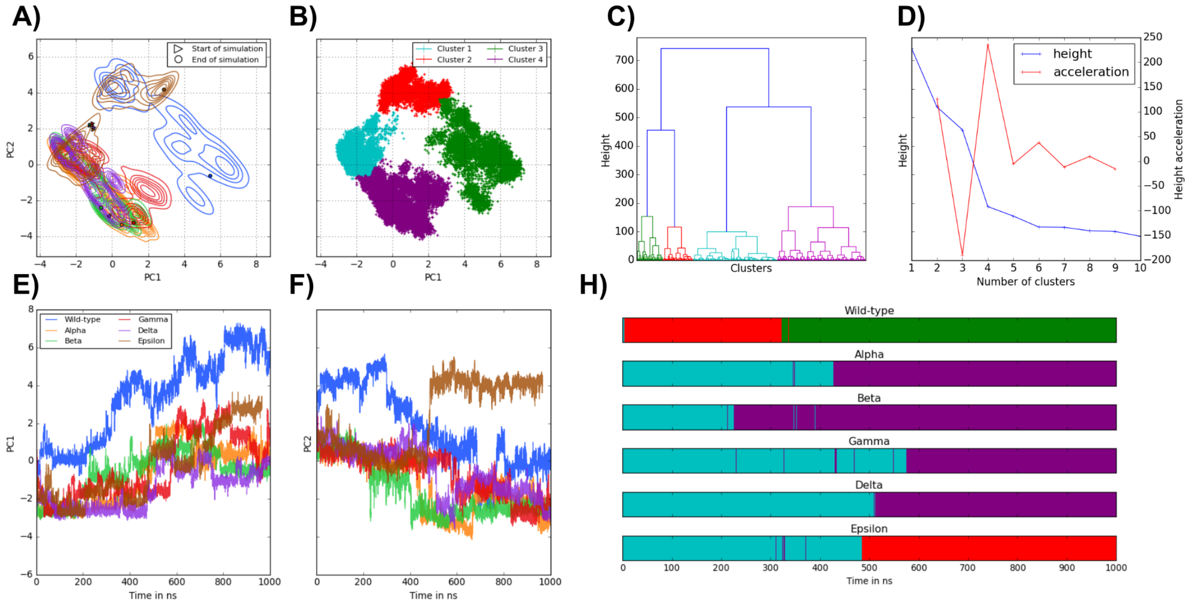

Figure S5. Projection of the frames corresponding to the microsecond simulations for the six studied complexes in the two dPCA eigenvector dimensions with (A) terrain lines representing a kernel density estimate of the population of each complex, (B) scatter plot representing the three main clusters obtained through Ward's minimum variance method. (C) Hierarchy obtained through Ward's minimum variance method and (D) acceleration plot displaying an optimal number of clusters equal to four. Time-plot of the (E) PC1 and (F) PC2 during each simulation. (H) Time evolution of each simulation in the different clusters.

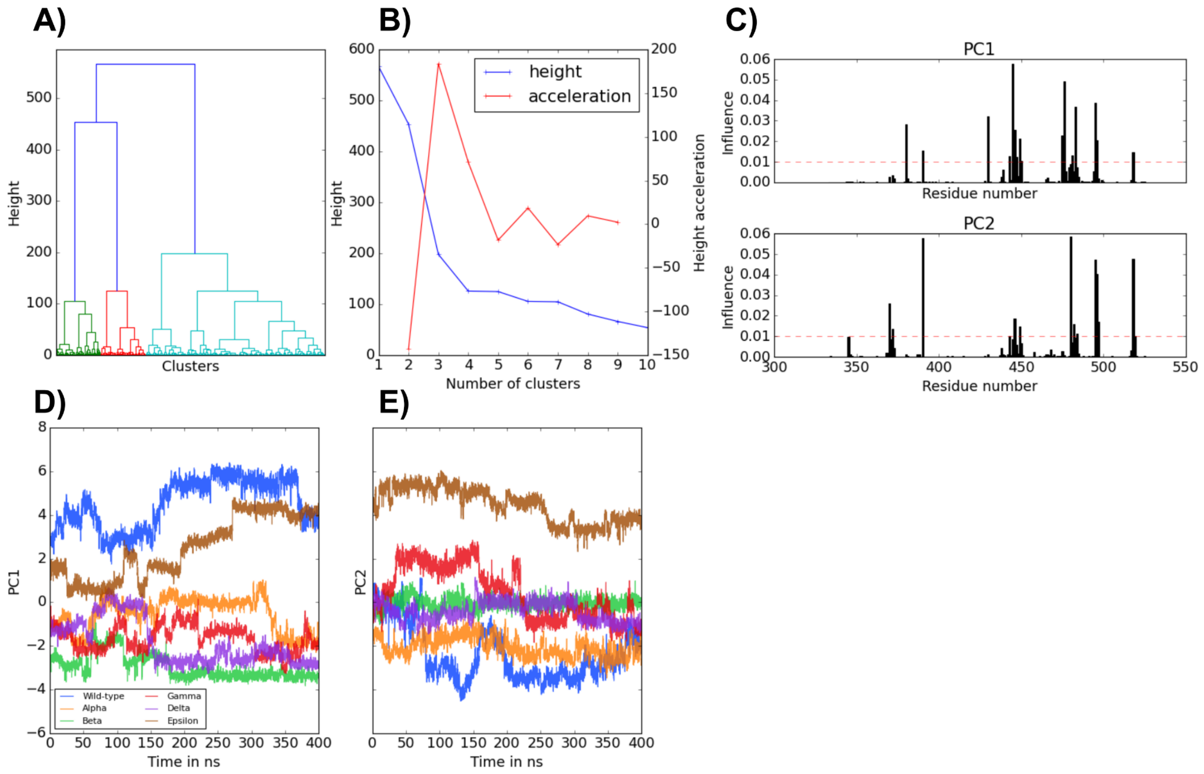

Figure S6. (A) Hierarchy obtained through Ward's minimum variance method and (B) acceleration plot displaying an optimal number of clusters equal to three. (C) Influence of each pair of consecutive residues in the PC1 and PC2. Time-plot of the (D) PC1 and (E) PC2 during each simulation.

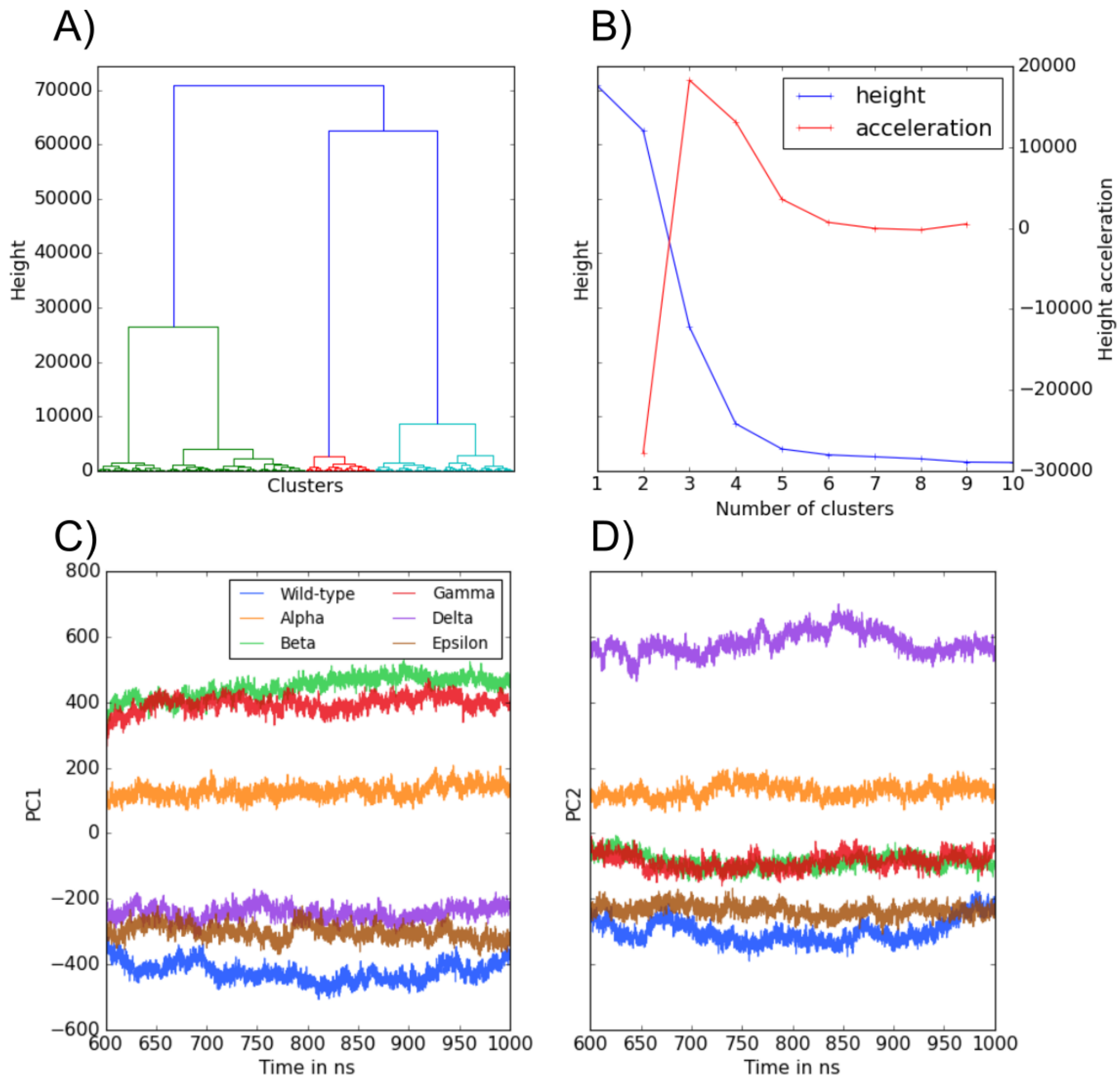

Figure S7. (A) Hierarchy obtained through Ward's minimum variance method and (B) acceleration plot displaying an optimal number of clusters equal to three. Time-plot of the PC1 (C) and PC2 (D) during the last 400 ns in each simulation.

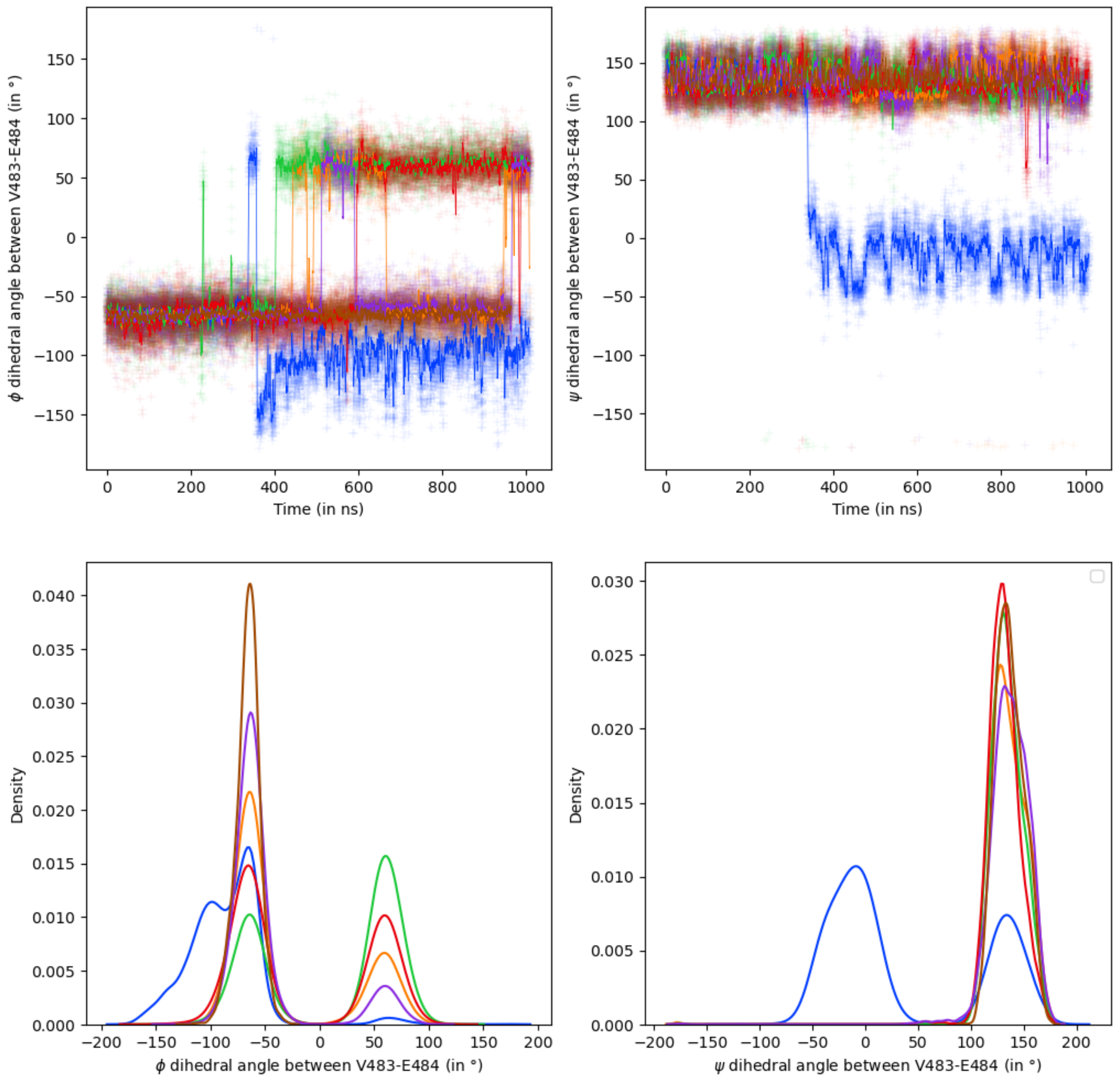

Figure S8. Time-evolution (left) and density (bottom) of the φ (left) and ψ (right) dihedral angles between V483 and E484.

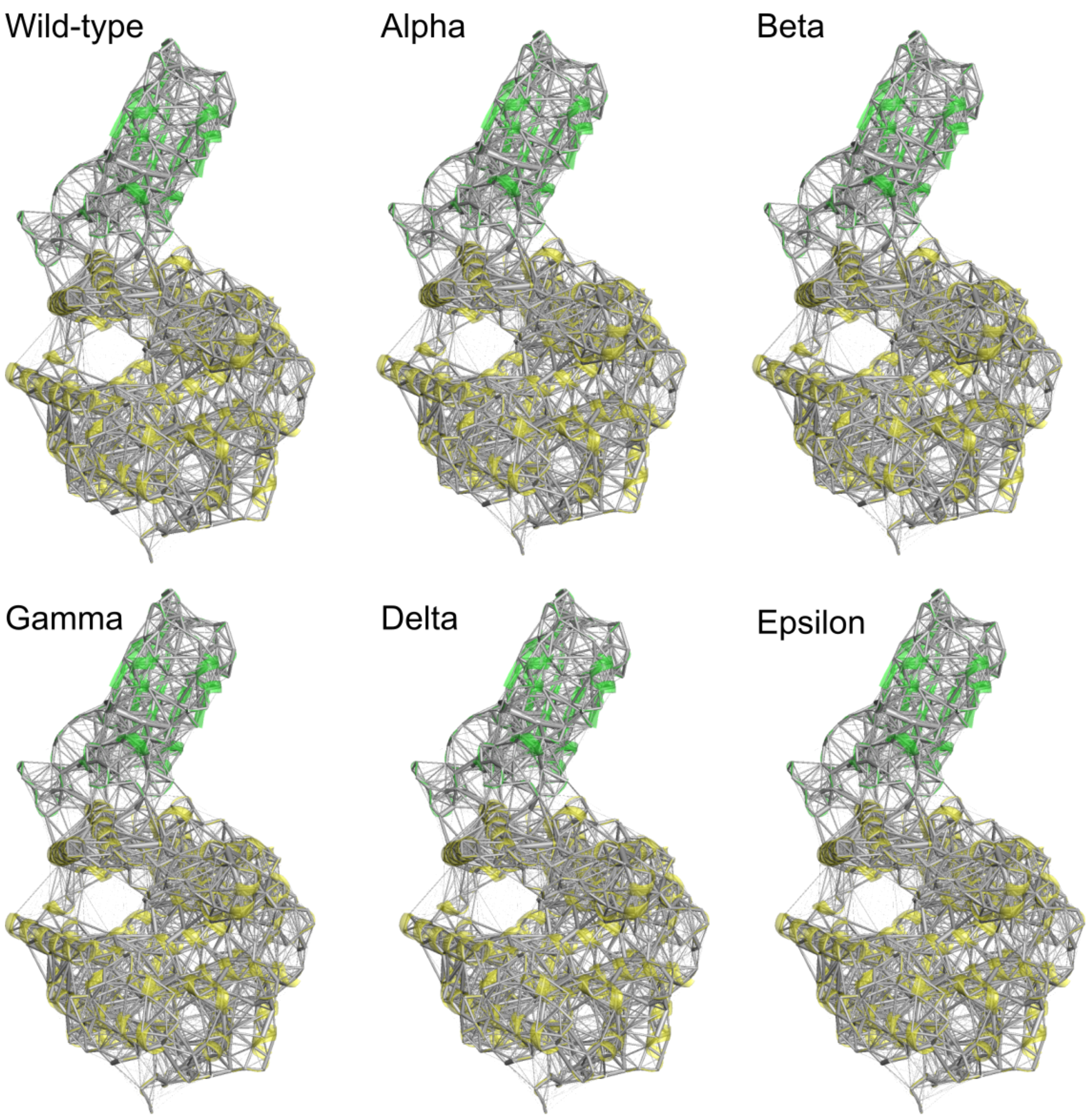

Figure S9. Complete contact network between the wild-type and each studied variant. The spike-RBD(green)/ACE2(yellow) complex is represented in cartoon representation. Contacts are represented with an edge width proportional to the number of interresidual atomic contacts.

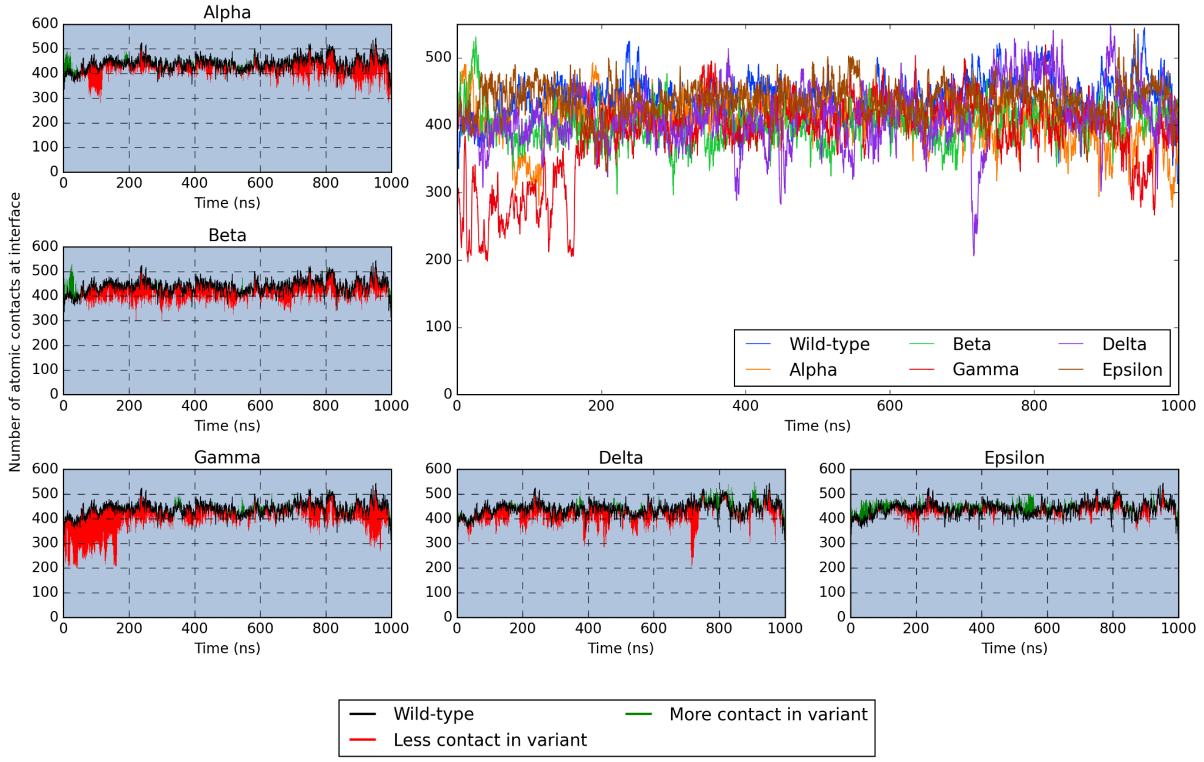

Figure S10. Number of heavy-atom contacts at the interface in function of the time for each simulation (top right panel). Individual comparison between each variant (in green if the number of contacts is bigger in the variant, red otherwise) and the wild-type (in black).

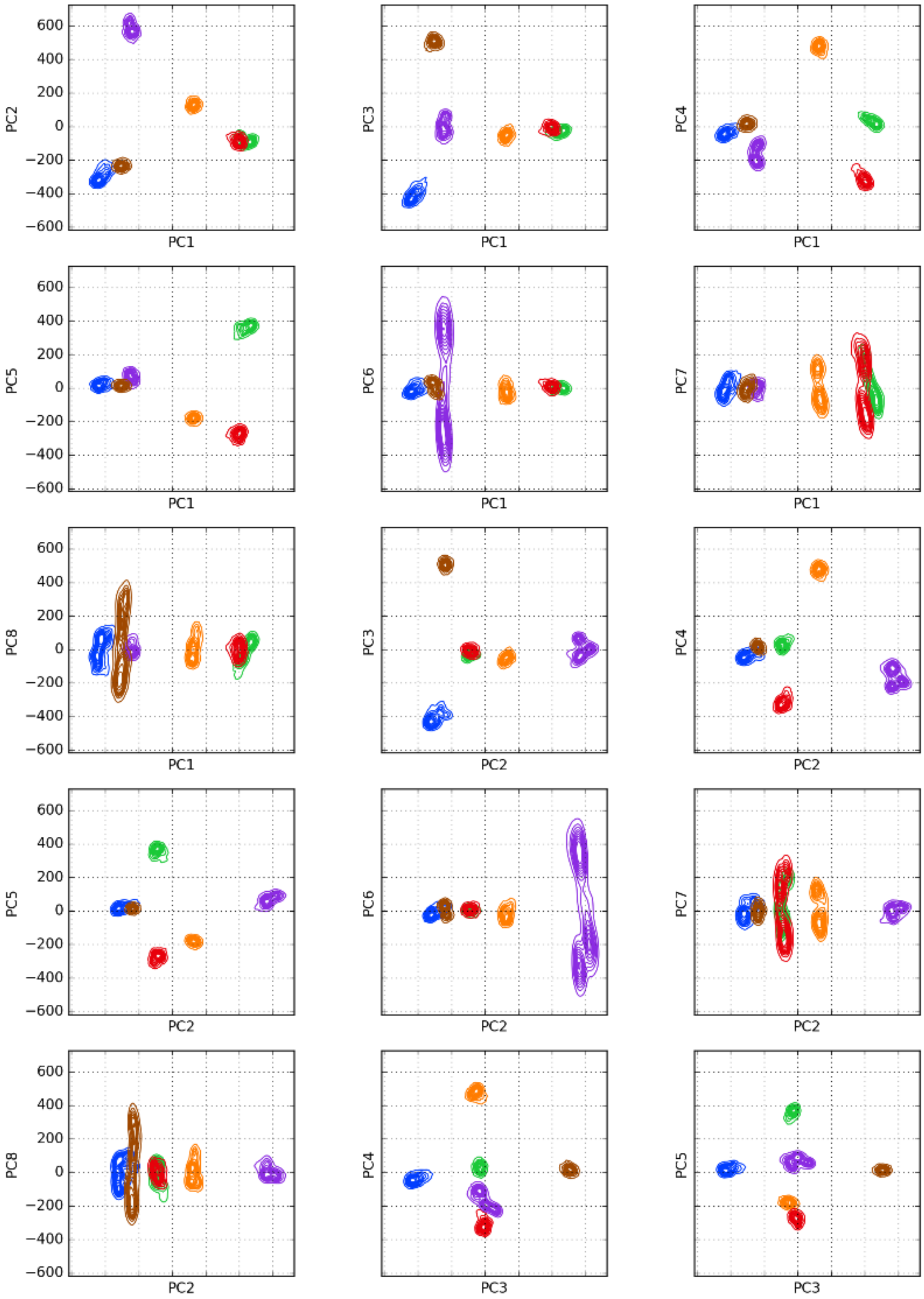

PC2
PC1
PC3
PC4
PC5
PC6
PC7
PC8
600
400
200
0
−200
−400
−600

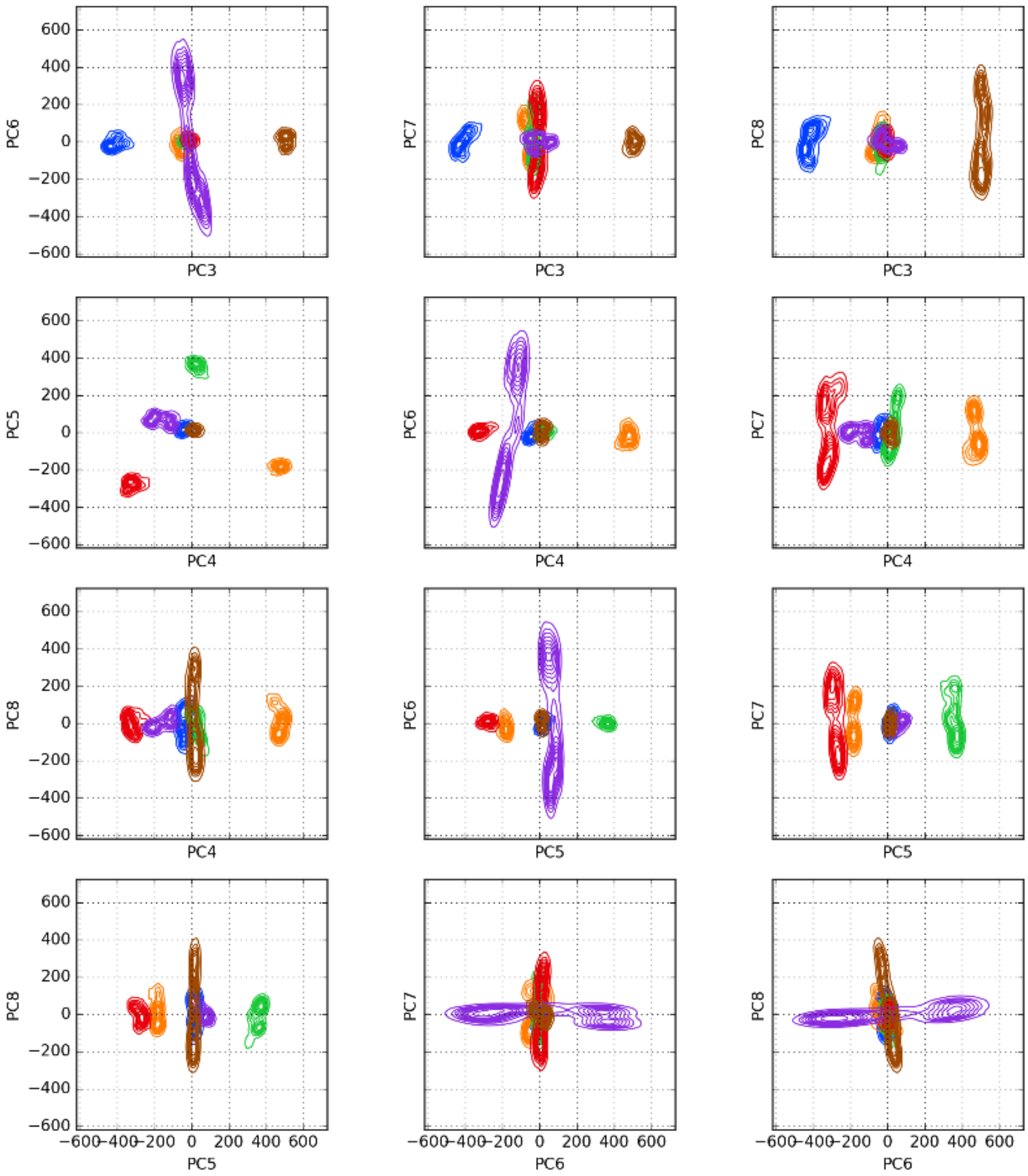

Figure S11. Free energy landscape associated with the different RBD variants in combination of the eight first cPCA eigenvector dimensions. Kernel density estimation shows regions with the highest population with terrain lines.

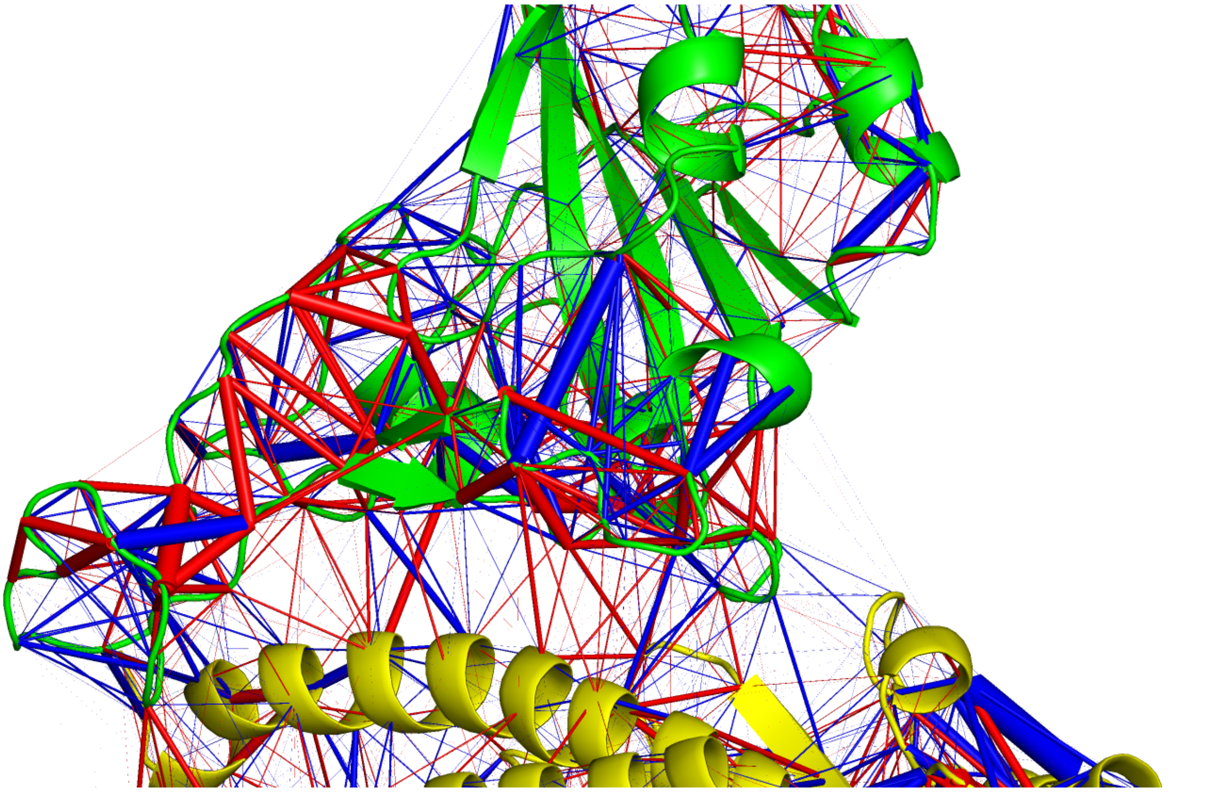

Figure S12. Eigenvector representation of the PC3 (a red edge means an increase in contact leads to positive values in PC3 and a blue edge means a decrease in this contact leads to negative values on PC3)

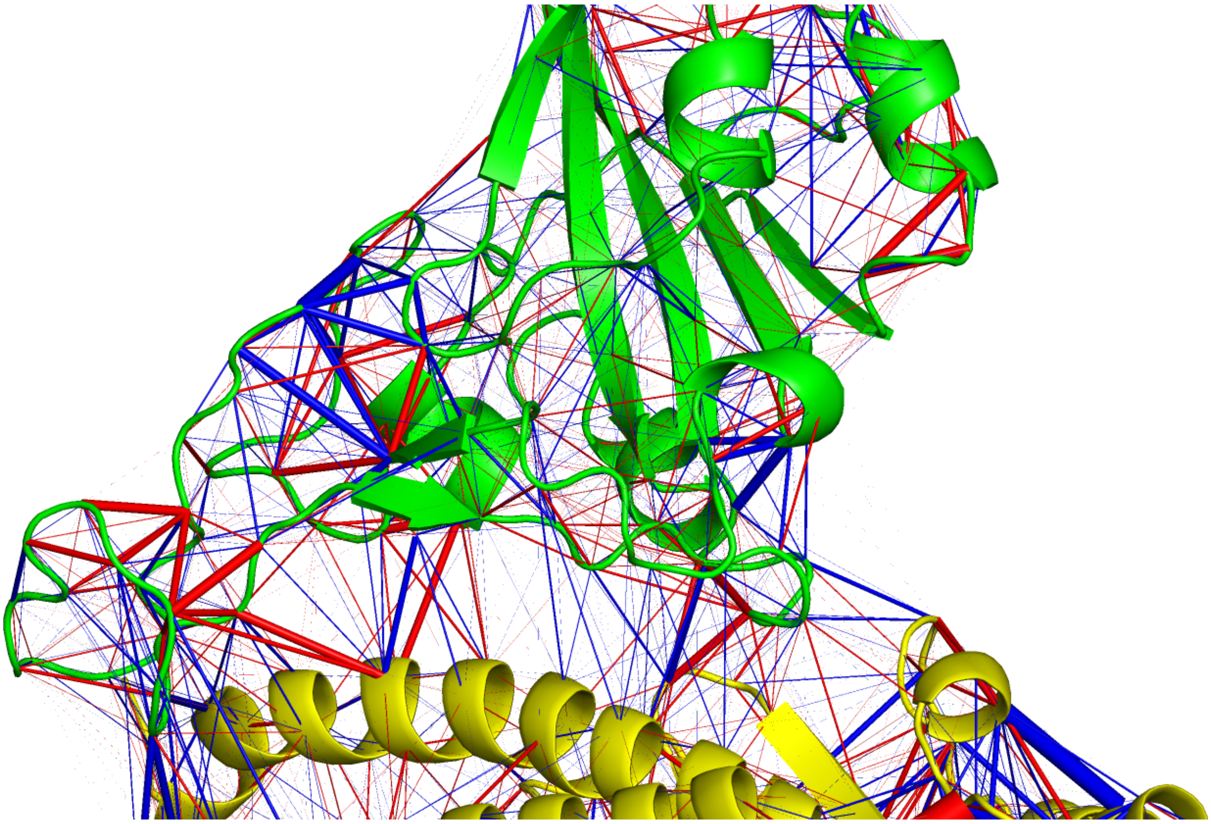

Figure S13. Eigenvector representation of the PC4 (a red edge means an increase in contact leads to positive values in PC4 and a blue edge means a decrease in this contact leads to negative values on PC4)

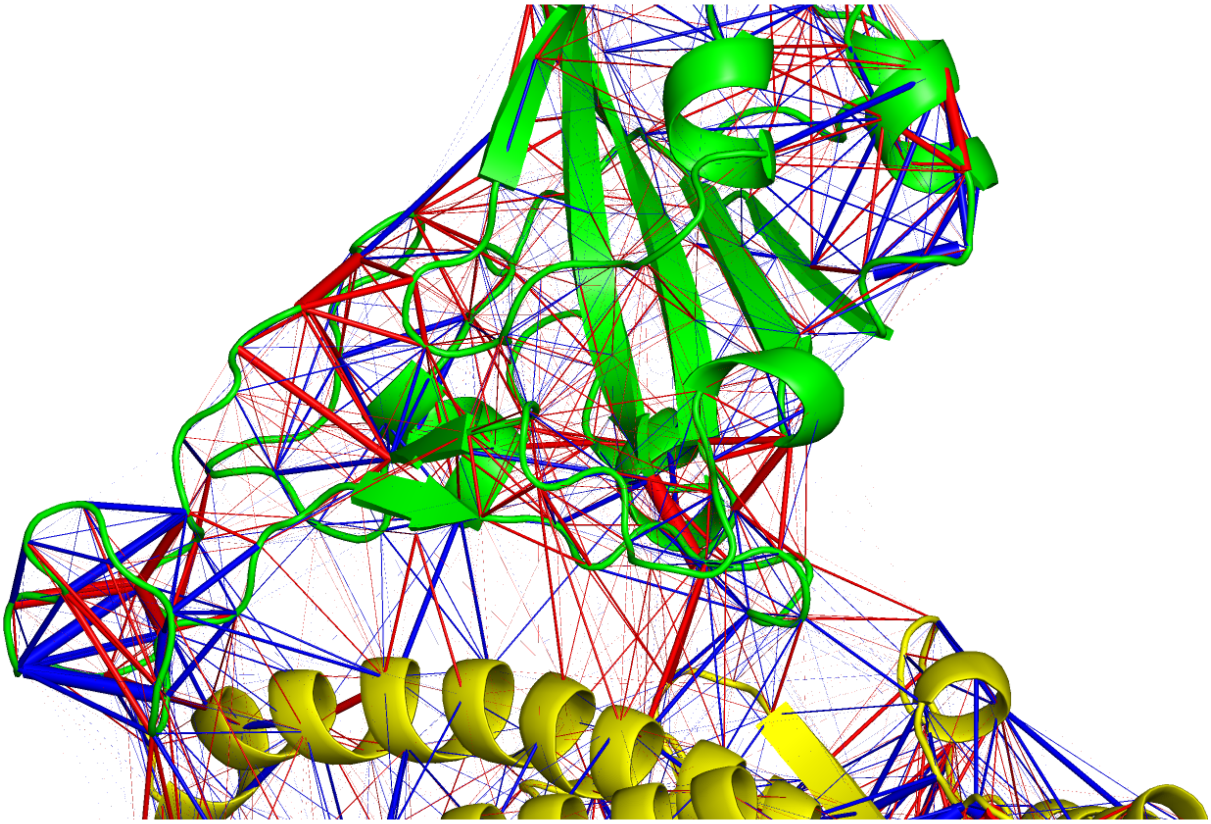

Figure S14. Eigenvector representation of the PC5 (a red edge means an increase in contact leads to positive values in PC5 and a blue edge means a decrease in this contact leads to negative values on PC5)

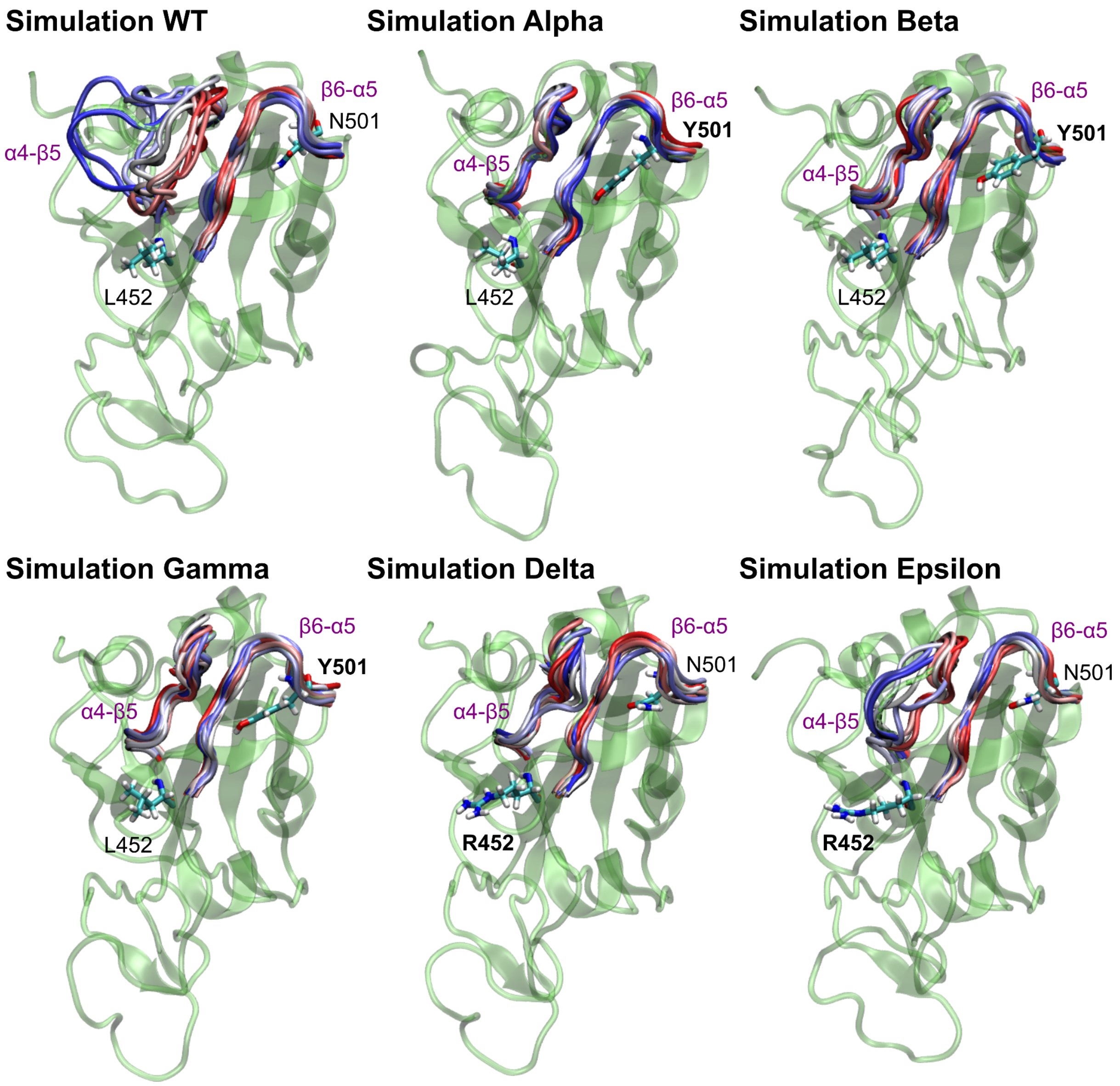

Figure S15. Spike RBD structure (in transparent lime) with the time-evolution of the α4-β5 and β6-α5 turn in contact (from the beginning of the simulation to the end from red to blue with structure printed each 100ns). The position of the mutated N501 and L452 residues are also shown in licorice.

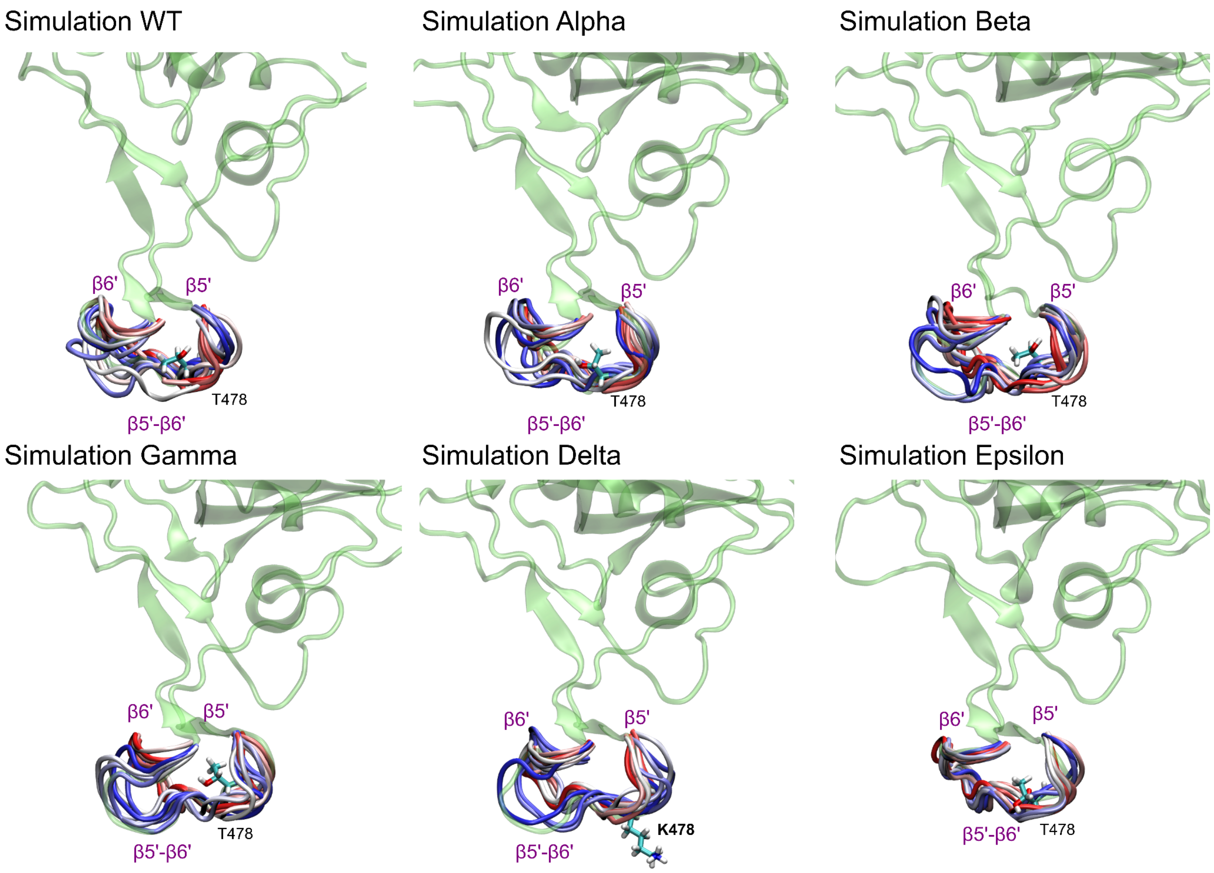

Figure S16. Spike RBD structure (in transparent lime) with the time-evolution of the β5'-β6' loop (from the beginning of the simulation to the end from red to blue with structure printed each 100ns). The position of the mutated T478 residue is also shown in licorice.
